# Proteomic Biomarkers Associated with Low Bone Mineral Density: A Systematic Review

**DOI:** 10.3390/ijms25147526

**Published:** 2024-07-09

**Authors:** Adriana Becerra-Cervera, Anna D. Argoty-Pantoja, Diana I. Aparicio-Bautista, Priscilla López-Montoya, Berenice Rivera-Paredez, Alberto Hidalgo-Bravo, Rafael Velázquez-Cruz

**Affiliations:** 1 Genomics of Bone Metabolism Laboratory, National Institute of Genomic Medicine (INMEGEN), Mexico City 14610, Mexico; abecerra@inmegen.edu.mx (A.B.-C.); daparicio@inmegen.gob.mx (D.I.A.-B.); priscilla_lopez92@comunidad.unam.mx (P.L.-M.); 2 National Council of Humanities, Science and Technology (CONAHCYT), Mexico City 03940, Mexico; 3 Research Center in Policies, Population and Health, School of Medicine, National Autonomous University of Mexico (UNAM), Mexico City 04510, Mexico; argotyanna@comunidad.unam.mx (A.D.A.-P.); bereriverap@comunidad.unam.mx (B.R.-P.); 4 Department of Genomic Medicine, National Institute of Rehabilitation, Mexico City 14389, Mexico; dr_genetica@yahoo.com

**Keywords:** proteomics, bone mineral density, biomarkers, osteoporosis

## Abstract

Osteoporosis is a globally relevant public health issue. Our study aimed to summarize the knowledge on the proteomic biomarkers for low bone mineral density over the last years. We conducted a systematic review following the PRISMA guidelines; the scoured databases were PubMed, Web of Sciences, Scopus, and EBSCO, from inception to 2 June 2023. A total of 610 relevant studies were identified and 33 were assessed for eligibility. Finally, 29 studies met the criteria for this systematic review. The risk of bias was evaluated using the Joanna Briggs Institute Critical Appraisal Checklist tool. From the studies selected, 154 proteins were associated with changes of bone mineral density, from which only 10 were reported in at least two articles. The protein–protein network analysis indicated potential biomarkers involved in the skeletal system, immune system process, regulation of protein metabolic process, regulation of signaling, transport, cellular component assembly, cell differentiation, hemostasis, and extracellular matrix organization. Mass spectrometry-based proteomic profiling has allowed the discovery of new biomarkers with diagnostic potential. However, it is necessary to compare and validate the potential biomarkers in different populations to determine their association with bone metabolism and evaluate their translation to the clinical management of osteoporosis.

## 1. Introduction

Osteoporosis is a systemic skeletal disease characterized by decreased bone mineral density (BMD) and an increased risk of fractures. Osteoporosis represents an economic and social burden worldwide; the main population affected is postmenopausal women [1]. The current standard tool for diagnosing osteoporosis is the measurement of BMD by dual-energy X-ray (DXA). However, DXA presents several challenges [2], such as the limited sensitivity in predicting fractures, the requirement for specialized facilities, associated high costs, the bulky nature of the equipment, and trained personnel [3], which become inaccessible in less-developed countries.

Many studies have also reported serum biochemical parameters, such as calcium, creatinine, and alkaline phosphatase, as predictors of osteoporosis [4,5,6]. However, they do not provide a clear pathway linking bone metabolism and are influenced by several exogenous factors [7]. Furthermore, previous studies had proposed several proteins as biomarkers for their direct (regulation of biological mineralization) or indirect (tissue remodeling and regulation immunity) biological effects. For example, osteopontin (OPN), a multifunctional phosphoprotein secreted by different cells such as chondrocytes, osteoblasts, and osteoclasts, is involved in bone strength and remodeling [8]. Clinical studies have shown that OPN could be targeted as a biomarker for early diagnosis of osteoporosis in postmenopausal women [9,10,11]. Another example is osteocalcin (OC), which is recognized as one factor synthesized and released by mature osteoblasts that could promote bone mineralization [12,13,14]. Therefore, serum OC levels may increase in postmenopausal osteoporosis [12,13], where a high bone turnover is present, with increased bone resorption.

Researchers have conducted proteome analysis of secreted proteins during osteoclast and osteoblast differentiation in the last 20 years to elucidate the molecular mechanism of bone remodeling [15,16,17]. Proteomics has been widely used during different stages of biomarker development: discovery, verification, and validation, in various pathologies, including osteoporosis [18,19]. Several important proteins related to BMD and osteoporosis have been identified using proteomic techniques. For instance, through the classical 2DE (two-dimensional gel electrophoresis) and 2D-DIGE (differential gel electrophoresis) coupled to mass spectrometry (2D-MS), researchers have found differentially expressed proteins in the blood serum of individuals with low BMD. Some of these proteins include Vitamin D Binding Protein (VDBP), ceruloplasmin (CP), and gelsolin (GSN), one of the proteins most frequently reported in patients with osteoporosis [20,21]. Additionally, label-free quantitative proteomics and techniques using multiplexing tags such as isobaric tags for relative and absolute quantitation (iTRAQ) and Tandem Mass Tag Reagents (TMT) have been employed for protein relative and absolute quantitation in large-scale proteomics studies, showing improved sensitivity and reproducibility over 2D-MS-based methods. Studies have shown that label-free quantification is commonly used to discover osteoporosis-associated biomarkers in human bone tissue, peripheral blood mononuclear cells (PBMCs), serum, and macrovesicles [22,23,24,25,26,27].

These techniques offer remarkable advantages, which could be used to assess the presence or progression of a disease and monitor response to treatment [28]. For example, a previous study validated seven proteins (IGHG2, C3, MEX3B, CRP, IGLC1, MYH14, and C1QC) as promising biomarkers for Saudi Arabian population suffering from osteoporosis or osteopenia [29]. In addition, an extended study profiled the serum proteome (*n* = 1785) of a Chinese population with 9.8 years of follow-up using liquid chromatography–tandem mass spectrometry (LC–MS/MS) [30]. They reported twelve proteins (PHLD, SAMP, PEDF, HPTR, APOA1, SHBG, CO6, A2MG, CBPN, RAIN, APOD, and THBG) associated with osteoporosis according to bone aging [30].

When paired with protein enrichment strategies, quantitative proteomics methods are powerful tools for identifying signaling molecules, modulators, and their interacting proteins in bone metabolism. This approach is essential for advancing our understanding of the molecular mechanisms underlying bone health and disease, potentially leading to the development of new therapeutic strategies for conditions such as osteoporosis.

Current evidence supports the importance of exploring proteomic approaches to identify differentially expressed proteins (DEPs) as potential biomarkers for predicting the development of bone diseases such as osteopenia and osteoporosis. This systematic review summarizes human studies focusing on the current knowledge about potential proteomic biomarkers related to low bone mineral density and identifies possible candidate proteins.

## 2. Materials and Methods

### 2.1. Search Strategy, Eligibility Criteria, and Study Selection

This study followed the Preferred Reporting Items for Systematic Reviews and Meta-Analyses (PRISMA) 2020 statement [31]. We conducted a systematic literature search using PubMed, Web of Science, Scopus, and EBSCO from inception to 2 June 2023. The following key terms were included in the search strategy: “proteome”; “proteomic”; “osteoporosis”; “osteopenia”; “bone mineral density”; “fracture”; “BMD”; “monocytes”; “serum”; and “plasma”.

Our inclusion criteria were the following: (1) observational studies (cohort, case-control, and cross-sectional studies) evaluating the association between proteomic markers and bone metabolism (osteoporosis, osteopenia, fracture, bone mineral density), (2) studies conducted in adults (aged ≥18 years), (3) studies written in English language, (4) studies reported as original research articles in peer-reviewed journals, and (5) full-text available. The exclusion criteria were: (1) studies in cell cultures or animals, (2) full-text version written in another language different to English, (3) review articles, letters to the editor, or case reports. These were seeded in text word searchers, and the “related articles” function was used to broad the search. We also reviewed publications cited in references using these search words for relevant studies that were not identified. In addition, all searches were conducted with no period specified. Concordance was evaluated through Fleiss’ kappa statistic. The protocol was registered in the International Prospective Register of Systematic Reviews (PROSPERO, ID: CRD42023431131).

### 2.2. Data Collection and Analysis

#### 2.2.1. Data Extraction and Management

Three researchers independently (P.L.-M., B.R.-P., and D.I.A.-B.) performed data extraction and were validated by independent researchers (A.B.-C. and A.D.A.-P.). The extracted information was added to a predetermined and standardized form using Microsoft Excel 365. Disagreements between researchers were discussed and resolved.

#### 2.2.2. Risk of Bias

The risk of bias of selected publications was evaluated using the Joanna Briggs Institute (JBI) Critical Appraisal Checklist, with a score of ≥5, 4, and <4 indicating low, moderate, and high risk of bias, respectively [32]. Three researchers (P.L.-M., B.R.-P., and D.I.A.-B) independently performed the risk of bias evaluation. An expert researcher (D.I.A.-B.) in proteomics solved disagreements between the researchers to establish the final selection of the articles to be included in this systematic review.

#### 2.2.3. Data Synthesis

The following information was extracted from the included studies extracted for detailed evaluation: basic information about the study (first author, year of publication, and country) and study population (country/ethnicity, sample size, sex, age, and outcome assessed). In addition, biological specimens collected, platforms used for proteomic analysis, and statistical analysis were also recorded. This review collected the proteins showing a significant change between conditions or those associated with abundance change in the low-BMD group during the discovery phase and the protein pathways related to bone loss.

#### 2.2.4. Network Analysis and Protein Enrichment

A protein–protein interaction (PPI) network was constructed with candidates from all articles using the online tool Searching The Retrieval of Interacting Genes/Proteins (STRING, https://string-db.org). The PPI network was constructed by setting medium confidence at 0.400.

To investigate the functions of potential biomarkers, the Gene Ontology (GO) term annotation was conducted by plug-in ClueGO (version 2.5.10) based on Cytoscape (version 3.10.1) [33]. GO terms were categorized into four modules: biological process, molecular functions, immune system, and cellular compartment localization.

## 3. Results

### 3.1. Systematic Research

**Figure 1:**
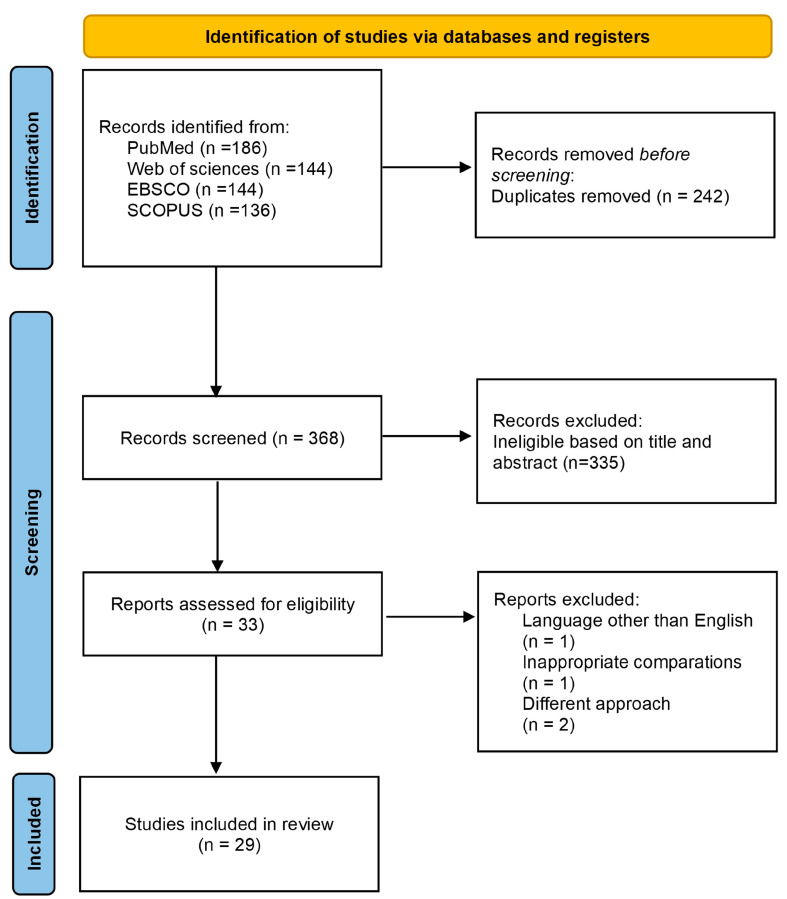
PRISMA 2020 flow chart describing the screening process.

The flow diagram of the literature search process is reported in Figure 1. The search strategy identified 610 relevant articles from PubMed, Web of Science, Scopus, and EBSCO. Data were exported to Excel, where 242 duplicates were removed. After reviewing the titles and abstracts, 335 were excluded, and 33 full-text articles were screened. From the 33 articles, four were removed, including where the technique did not identify specific proteins (*n* = 2), a full-text version was written in another language (*n* = 1), and there were inappropriate comparisons (*n* = 1). Finally, 29 articles meeting the criteria were included in this systematic review. An agreement percentage of 97% (Fleiss’ kappa = 0.83, *p* < 0.001) was observed between reviewers.

### 3.2. Study Characteristics

The characteristics of the 29 original studies included are summarized in Table 1. Among the selected studies, the most common ethnic group analyzed was Chinese (*n* = 14) [26,34,35,36,37,38,39,40,41,42,43,44,45,46], followed by Caucasian (*n* = 6) [23,24,25,47,48,49], and Italian (*n* = 2) [50,51]. The rest of the studies analyzed the following populations: Indian (*n* = 1) [22], Croatian (*n* = 1) [52], Saudi Arabian (*n* = 1) [29], non-Hispanic white (*n* = 1) [27], Swedish (*n* = 1) [53], and Mexican-Mestizo (*n* = 1) [20]. One study did not report ethnicity [54].

Overall, samples from 3538 subjects (cases and controls) were used for the biomarker discovery phase alone. The cases included in the studies ranged from 4 to 237 patients, with a median of 20. The age range in osteoporotic patients was from 55.2 to 81 years, while for individuals classified as low-BMD (LBMD) it was from 36 to 70 years old [52,53]. Approximately half of the studies were conducted exclusively in women, either pre- (*n* = 4) or postmenopausal (*n* = 14); the remaining studies included both male and female adults.

Overall, 86% were case-control studies, 10% were cross-sectional, and 4% were cohort studies. All studies measured the BMD by DXA, except one where urine N-telopeptide of type I collagen scores were used [27]. Most of the included studies applied the World Health Organization’s (WHO’s) criteria for diagnosis of osteopenia or osteoporosis, followed by BMD criteria adjusted by ethnicity population; only one study did not report the BMD criteria. Nine studies (56%) analyzed the proteome in both osteopenia and osteoporosis, five studies (31%) focused exclusively on osteoporosis, and two studies (13%) evaluated only osteopenia. Notably, twenty-three (79%) of the reports were adjusted by confounding variables, mainly age, weight, height, or body mass index (BMI), while the rest did not report any adjustment.

### 3.3. Proteomic Techniques

**Figure 2:**
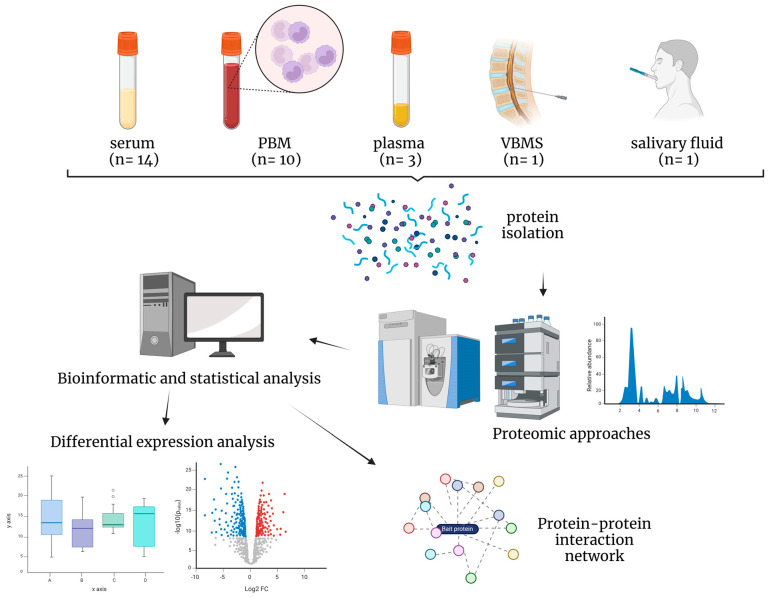
Schematic diagram of summary of biological samples analyzed among studies. PBM: peripheral blood monocytes, VBMS: vertebral body-derived bone marrow supernatant fluid. Created with BioRender.com (accessed on 1 March 2024).

The source of proteins for biomarker discovery included serum (*n* = 14), peripheral blood monocytes (PBM) (*n* = 10), plasma (*n* = 3), vertebral body-derived bone marrow supernatant fluid (*n* = 1), and salivary fluid (*n* = 1) (Figure 2). Three of the fourteen serum studies were directed to exosomes [34,40] and one to microvesicles [26]. Details of proteomics analysis are shown in Table 2 and Table S1. The most common proteomic approach employed was Nano-LC-ESI-MS, used in seven studies, followed by TMT-LC-MS, with four studies (Table 2). Regarding the sample type and data treatment, 42% of the studies used several strategies for depleting highly abundant plasma proteins (albumin and immunoglobulins) and enriching low-abundant proteins. Multiple statistical analyses were performed among proteomic studies to select the potential biomarkers. Seven reports revealed a strict eligibility criterion for biomarkers, mainly fold change (FC) ≥ 2 or 1.5, where upregulation was ≥2 or 1.5 times or downregulation ≤0.5 or 0.6 times and *p*-value < 0.05, indicating a statistically significant difference in the DEPs. In fifteen articles (51%), potential biomarkers were validated after the proteomic approach employed techniques were, Western blot (WB) (*n* = 5), ELISA (*n* = 7), and parallel reaction monitoring analysis (PRM) (*n* = 3) (Table S2).

### 3.4. Main Studies Performed

Examining the selected studies, the identified DEPs varied between 4 and 294, particularly when comparing the osteoporotic and normal groups. Overall, 154 unique proteins were reported in the discovery phase as potential biomarkers altered in LBMD individuals. Ten studies were conducted in serum, enabling the identification of 41 DEPs. The proteomic profiling of exosomes and serum microvesicles showed eight and three biomarkers, respectively. Three studies focused on plasma samples, identifying 13 DEPs. Further, in the study that used an antibody array, seven DEPs were identified in plasma comparing OP vs. N. A total of 79 potential biomarkers were identified among studies where PBM were used (*n* = 10), comparing LMBD vs. high-BMD (HBMD). A study reported 45 DEPs comparing the proteome of pre- vs. postmenopausal groups. In addition, six proteins were identified in vertebral body-derived bone marrow supernatant fluid, ten in extracellular vesicles (EVB), and one in salivary fluid. Four studies have characterized the proteomic profiling in men with high and low hip BMD [25,27,43,49]. Seven studies were conducted in samples of PBMs derived from women aged between 27 and 55 years, classified as extremely LBMD and HBMD [22,24,35,45,46,48,52]. Five studies were conducted comprising women and men. Among fourteen studies conducted in postmenopausal women, seven were performed in serum [20,37,38,39,42,44,54], three in PBMs [22,23,47], two in plasma [36,53], one in extracellular vesicles blood [50], and one in salivary fluid [51].

### 3.5. Risk of Bias

The risk of bias was low when assessing the entire set of studies (Tables S3–S5).

### 3.6. Potential Protein Biomarkers Found in Two or More Studies

As shown in Table 3, ten DEPs were observed in at least two studies.

### 3.7. Pathways

**Figure 3:**
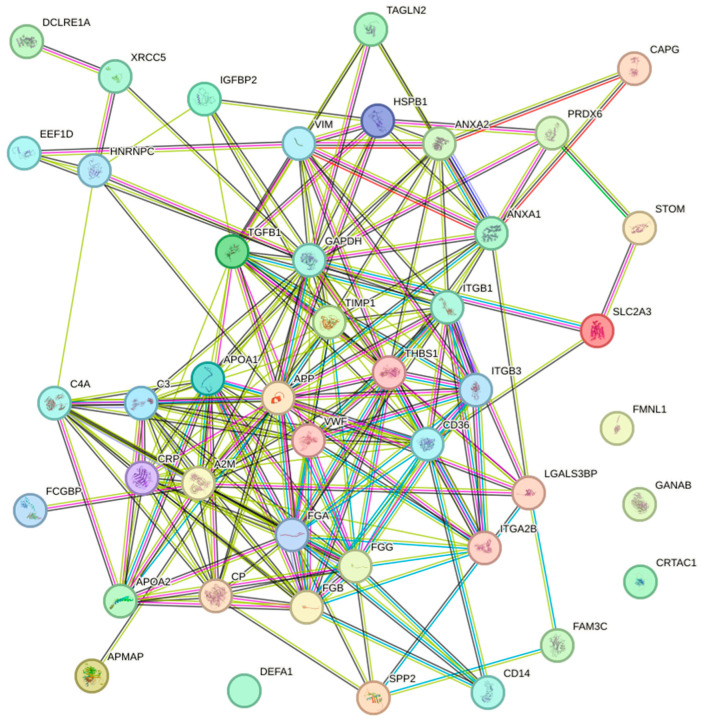
The subnetwork of DEPs associated with the skeletal system visualized by STRING.

A protein–protein interaction (PPI) network was constructed using the STRING database. A total of 159 different DEPs were found in all the selected studies; however, the network excluded 14 DEPs (CFD, IGKC, IGVL, IGKV2DM, B7Z795, IGHG2, IGLC1, RMCX3, GPX1, DKFZp666N164, B4DE30, PPIAP19, LOC388720, and ANXA2P2) due to the lack of information. The obtained network comprised 136 nodes and 670 edges with a *p*-value < 1.0 × 10^−16^. A subnetwork associated with the skeletal system (BTO: 0001486) was detected; it contained 44 nodes and 206 edges with a *p*-value < 1.0 × 10^−16^ (Figure 3, Table S5). Additionally, the hemostasis (HSA-109582) and extracellular matrix organization (HSA-1474244) pathways were also identified. Most of the proteins were associated with biological process, including the immune system process (GO: 0002376), regulation of protein metabolic process (GO: 0051246), regulation of signaling (GO: 0023051), transport (GO: 0006810), cellular component assembly (GO: 0022607), and cellular differentiation (GO: 0030154) (Table S5).

**Figure 4:**
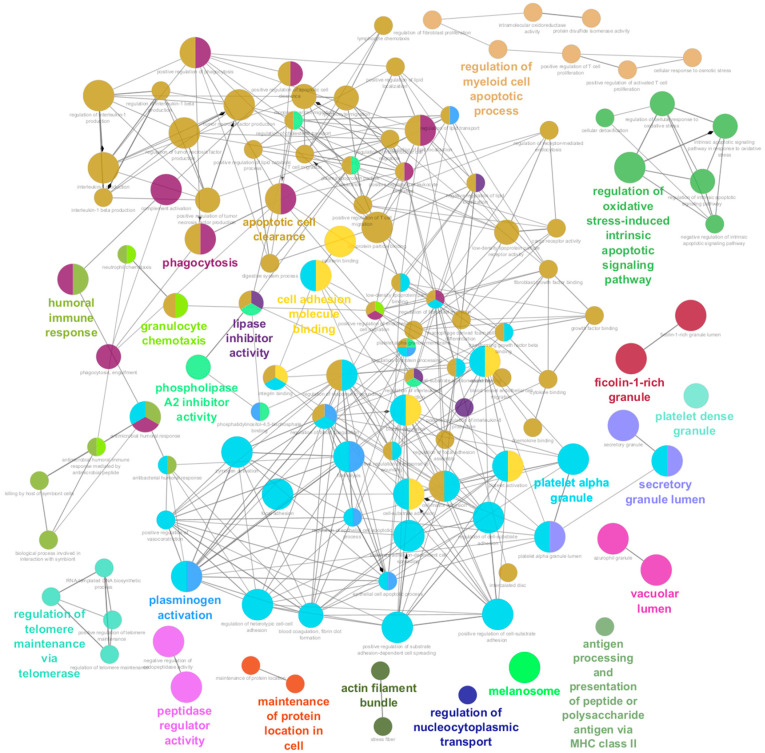
Enrichment by pathways is visualized using the ClueGo plugin from Cytoscape. The plugin shows the main enrichment pathways in the three modules of KEGG using the protein biomarker found in the discovery phase. See detailed pathways in Supplementary Table S7.

To better understand the biological function of the reported biomarkers, the plug-in ClueGO [33] was used to generate network showing the interconnection between enriched pathways. The Gene Ontology (GO) enrichment of differentially expressed proteins were performed with statistical criteria set at *p* ≤ 0.05, and the pathway terms were ranked based on the fold enrichment. Pathway enrichment using the 159 DEPs as input revealed that they participate in 174 pathways. In order to reduce redundancy, the obtained pathways were regrouped into 22 using the GO term fusion. Details of the enriched pathways are presented in Table S6. The top enriched pathways were the secretory granule (GO: 0030141), lumen (GO: 0034774), and focal adhesion (GO: 0005925). Following the cell adhesion molecule binding and focal adhesion in molecular function. Among biological processes, pathways included wound healing, phagocytosis, and cell-substrate adhesion (Figure 4).

## 4. Discussion

Proteomic analyses are becoming a powerful approach for identifying key players participating in the loss of bone mass. Herein, we conducted a systematic review to investigate potential proteomic biomarkers for early detection of bone loss. Most of the reviewed studies were conducted in Asian populations under a case-control design. Proteins in blood samples (serum, plasma, and PBMs) were the most analyzed as promising biomarkers. Findings were categorized according to the population studied. From the 159 DEPs reported in the selected studies, ten were identified in at least two studies in LBMD patients. Based on this criterion our discussion is focused on these ten proteins (Table 3).

**Figure 5:**
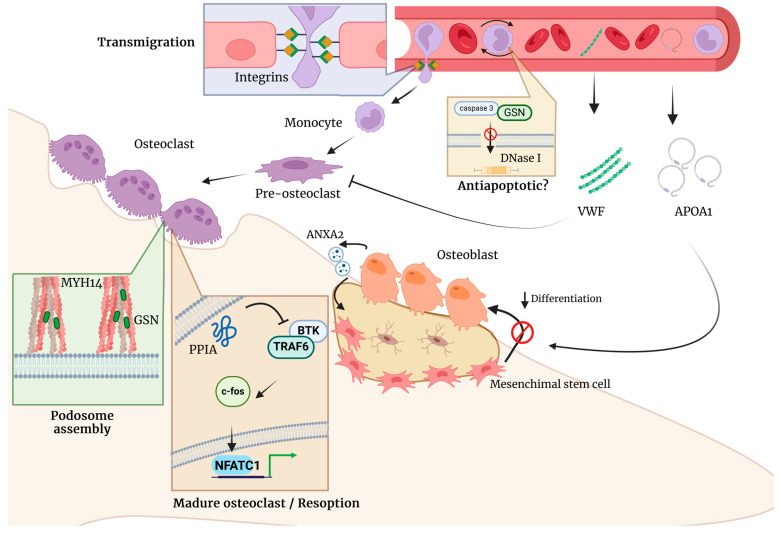
Schematic diagram of the mechanism of differential expressed proteins associated to changes of bone mineral density. Squares indicate different pathways. APOA1: Apolipoprotein AI; ANXA2: Annexin A2; GSN: Gelsolin; MYH14: Myosin heavy chain 14; PPIA: Peptidyl-prolyl cis-trans isomerase A; VWF: Von Willebrand factor. Created with BioRender.com (accessed on 1 March 2024).

Gelsolin (GSN) is a calcium-dependent actin-binding protein and plays an important role in cell mobility, cell shape, actin cytoskeleton, regulation of cell signal transduction, metabolic processes, and apoptosis [55]. GSN expression levels have been related to osteoporosis [56]. Gelsolin-deficient mice block osteoclast podosome assembly and motility-related αvβ3-stimulated signaling, thereby developing thicker, fracture-resistant cortical and trabecular bone while decreasing rates of bone resorption (Figure 5) [57]. The relationship between GSN and BMD has been consistent. Increased GSN levels in serum and plasma have been associated with LBMD in postmenopausal women [20,48]. These findings suggest that GSN promotes osteoclastogenesis and bone resorption by enhancing osteoclast migration, adhesion, and activity (Figure 5) [35]. However, higher levels of gelsolin expression in PBM may promote growth inhibition and pro-apoptosis of monocytes, reducing osteoclast formation and bone resorption, therefore increasing bone mass [47]. Overall, GSN is the main protein pointed out as a potential biomarker for osteoporosis. Further studies are needed to clarify the role of GSN in BMD.

Annexin A2 (ANXA2), a class of calcium-dependent phospholipid-binding proteins, has been reported to be involved in multiple cellular processes, such as proliferation, apoptosis, and migration [58]. ANXA2 has the critical role of initiating the mineralization process in cartilage, while in bone, it is proposed to participate in the influx of Ca^2+^ into the matrix vesicles [59]. The proteomic approaches have revealed that ANXA2 was significantly upregulated in PBM samples from Caucasian postmenopausal women with LBMD. At the same time, a report showed that levels of ANXA2 were considerably decreased in Indian postmenopausal women with LBMD. In addition, Deng et al. [47] reported that extracellular ANXA2 promotes monocyte migration across the endothelial barrier in vitro (Figure 5), which probably increases the number of osteoclasts. Thus, it could encourage bone resorption at higher rates, thereby decreasing BMD. They also reported upregulation of the ANXA2 gene in PBM derived from LBMD individuals [47]. Although previous evidence supports a significant role for ANXA2 protein in bone remodeling, it requires further investigations in other populations.

Von Willebrand factor (VWF) is a multimeric glycoprotein mainly expressed in endothelial cells and megakaryocytes with a primary function in hemostasis [60]. Previous studies in animal models have shown bone loss in the presence of coagulation factor deficiencies [61]. According to the selected studies, VWF was identified as differentially expressed in serum and extracellular vesicles, although the results are contradictory [37,50]. VWF was significantly upregulated in postmenopausal osteoporotic women [37], whereas it is absent in extracellular vesicles from patients with LBMD [50]. VWF participates indirectly in the maintenance of bone, the FVIII-VWF complex can inhibit RANKL-induced osteoclastogenesis by binding to RANKL. In addition, the FVIII-VWF complex inhibits osteoprotegerin (OPG) [62], a glycoprotein that regulates bone resorption (Figure 5) [63]. These interactions between the FVIII-VWF complex, OPG, and RANKL increase the anti-osteoclastic activity of OPG, which contributes to the homeostasis of bone. Nevertheless, additional studies must demonstrate its role in physiological bone remodeling or damage.

On the other hand, Protein disulfide-isomerase (P4HB) is a key enzyme for protein folding, as it forms the correct disulfide bridges between polypeptide chains and regulates apoptosis [64]. P4HB has been reported to be a novel candidate gene for a severe type of osteogenesis imperfecta [65]. A heterozygous missense mutation in exon 9 of P4HB, located in the C-terminal, sterically close to the catalytic site affects the disulfide isomerase activity in vitro, generating severe bone fragility [66,67]. In addition, a decreased protein expression has been observed in patients with LBMD [35]. However, contradictory data have been reported [49]. Given these results, additional studies in other populations must confirm or rule out its role as a potential biomarker for LBMD.

Integrins, a family of heterodimeric transmembrane glycoproteins, are recognized for their role in mediating cell–cell and cell–matrix interactions [68]. Investigations into the role of integrins in bone homeostasis have revealed intricate insights. Firstly, in premenopausal Caucasian women, integrin subunit alpha 2B (ITGA2B) did not exhibit a significant association with hip BMD. In osteoporosis, the downregulation of integrin receptors α1, β1, and β3 in serum-derived exosomes (SDEs) affects cell adhesion. This observation suggests a dysregulation of the P13K/AKT pathways, potentially hindering osteoblast function and impairing mineralization. In contrast, osteopenia SDEs show a slight upregulation of integrin-mediated proteins, differing from osteoporosis. The downregulation of TGF-β pathway proteins in osteoporosis may disrupt bone remodeling through SMAD proteins. SDEs from osteoporosis and osteopenia patients promote osteoclast formation and bone resorption, suggesting a role in modulating osteoclast activity [40].

Additionally, Zeng et al. provided valuable insights into integrin regulation in bone homeostasis, strengthening the association of ITGA2B with osteoporosis predisposition and highlighting its role in critical pathways. These findings underscore the pivotal roles of these genes in bone-related pathologies [24]. In a parallel study on male subjects, DEPs were identified in monocyte membrane components and Integrin b1 (ITGB1) was found, shedding light on the possible contribution to osteoporosis. Functional analysis revealed their enrichment in crucial pathways for bone metabolism, such as “ECM receptor interaction” and “leukocyte transendothelial migration” [49]. The contradictory results can arise from various factors, including the complexity of genetic contributions to BMD regulation, the potential influence of other unexplored genetic or environmental factors, and inherent heterogeneity within study populations (Figure 5) [48]. However, the evidence indicates that the roles of ITGA2B and ITGB1 in bone metabolism need to be investigated in other populations.

Myosin heavy chain 14 (MYH14) belongs to the family of ubiquitous actin-based motor proteins involved in cytokinesis, vesicular transport, and cellular locomotion in eukaryotic cells. In two studies, the expression levels of MYH14 showed significant upregulation in Caucasian men and Saudi Arabian women and men with LBMD compared to HBMD subjects [25,29]. In addition, Al-Ansari et al. [29] reported a linear increase in the levels of this protein across the control, OS, and OP groups. Previous studies have shown that actin-based motor-like proteins, such as MYH14, regulate osteoclast migration, tunneling nanotube formation, and actin organization necessary for osteoclast fusion (Figure 5) [69]. Furthermore, the MYH14 gene was associated with BMD in multiple omics studies (transcriptomic and genomic) [25]. Although its role in bone physiology has not been studied, MYH14 could play a role in osteoclast podosome formation and bone resorption.

Two studies included in this review reported increased levels of Apolipoprotein A-I (APOA1) in serum and plasmatic extracellular vesicles of postmenopausal women with OP [42,50]. The APOA1 knockout mice model revealed that in the absence of this apolipoprotein, the mesenchymal stem cells differentiation shifts towards lipoblasts precursor cells with reduced osteoblast development without affecting the osteoclast production (Figure 5) [70]. In addition, other recently published articles reported variation in APOA1 levels. Nevertheless, results are contradictory [23,30,50]. It should be noted that the subjects evaluated in these proteomic approaches are from different ethnicities, and patients with metabolic diseases related to lipid dysregulation were always excluded. However, these reports suggest an important relationship between APOA1 and BMD that requires a deeper functional characterization.

Reduced BMD can be caused by decreased osteoblast activity, often accompanied by increased osteoclast function. In this regard, the enzyme Peptidyl-propyl-cis-trans isomerase A (PPIA) has been reported as a critical dual regulator of bone anabolism and resorption. Guo and et al. [71] described that PPIA is required for osteoblast differentiation through BMP-2-induced Smad1/5/8 phosphorylation for regulating Runx2 activation. On the other hand, RANKL-induced osteoclastogenesis is interrupted as PPIA hinders BTK phosphorylation and disrupts NFATc1 expression (Figure 5) [72]. In addition, PPIA has been consistently identified in PBM samples from Caucasian postmenopausal women. In a discovery phase, PPIA was significantly upregulated in a group with extreme HBMD [47]. In another report, it was significantly downregulated in the LBMD group [23]. Furthermore, gene set enrichment analysis revealed enrichment of the “platelet activation, signaling, and aggregation” and “homeostasis” pathways related to PPIA [23].

According to evidence, GSN is the most frequently reported protein as a potential biomarker in the discovered stage, followed by ANXA2 and APOA1. These data are consistent with previous proteomics studies, where GSN levels were negatively correlated with total hip BMD in Caucasian and Mexican postmenopausal women [20,24,48]. In contrast, a study on Chinese women showed that GSN was upregulated in the LBMD group [35]. Meanwhile, ANXA2 and APOA1 showed inconsistencies in direction regulation. Several factors may influence these results and should be considered in future studies. These experimental designs include true analytical variability in clinical samples, such as age, which, in these studies, postmenopausal women are more susceptible or exposed to than other age groups. A possible explanation for contradictory results by ANXA2 and APOA1 could be attributable to ethnicity background, as previously it has been reported in various diseases [73,74]. Nonetheless, research into race and ethnicity remains controversial, with some questioning its utility in clinical practice. Additionally, some variation may be due to lifestyle factors and environmental and temporal variability. Thereby, studies based on patient samples could consider the confounding factors on protein level.

On the other hand, the independent enrichment analysis performed in STRING and ClueGO confirmed the extensive role of proteins associated with bone remodeling, for instance, immune system-related processes (such as humoral immune response and antigen processing, presentation of peptide and polysaccharide antigen via MHC class II), cellular component assembly-related process (actin filament bundle, cell adhesion molecule binding, phagocytosis, vacuolar lumen, secretory granule lumen, and extracellular matrix organization), and regulation of metabolic process. Among DEPs, ANXA2 and APOA1 were the proteins presenting most interactions between pathways, suggesting that they may play an essential role in bone loss. The ITGA2B also clustered with processes related to the skeletal system, such as cadherin binding and cell-matrix adhesion, whereas ITGB1, PPIA, and GSN participate in the cell differentiation and assembly in the immune system or osteoclast migration, as it has been described before for GSN.

The immune system’s role in BMD has been widely associated with the remodeling and integrity of bone [75,76,77,78]. Several studies have postulated the relationship between systems, where the activation of some types of immune cells could promote the maintenance of bone mass [30,76], e.g., CD8+ T cells have been recently related to bone-protecting functions through the secretion of osteoprotegerin and interferon (IFN)-γ [79,80]. In contrast, the upregulation of the via of receptor activator of NF-κB by IL-17 induces higher levels of RANK ligand in osteocytes, leading to osteoclastogenesis [81]. Additionally, in postmenopausal women with estrogen deficiency, the dysregulation of the immune system reduces the osteoclastic effects and induces osteoclast apoptosis [82]. Thus, the immune system remodeling characteristic of aging is a determining factor associated with the etiopathogenesis of osteoporosis that undoubtedly may be the basis of future research as a novel therapeutic tool in osteoporosis.

Not surprisingly, DEPs participate in cellular component assembly-related processes, including actin filament bundle, cell adhesion molecule binding, and extracellular matrix organization. Among these proteins, GSN and ANXA2 have been demonstrated to be involved in remodeling bone [47,57,58]. Furthermore, integrin adhesions are necessary for podosomes, specialized cell surface structures actively involved in bone degradation [83]. In osteoclasts, the podosomes seal the gap between the ventral membrane and the bone surface and secrete protons and proteases into the gap [84], thereby supporting the resorption of the underlying bone.

Another pathway involved in the metabolic processes is enzymatic activity, which extends to clinical translation for inhibiting lipase and phospholipase activity as a novel anabolic therapy. For instance, Monoacylglycerol lipase (MAGL), a lipolytic enzyme that catalyzes monoglycerides hydrolysis, shows increased expression during osteoclast differentiation. Thus, pharmacological inhibition of MAGL by JZL184 suppressed osteoclast differentiation, bone resorption, and osteoclast-specific gene expression. Activation of the mitogen-activated protein kinase (MAPK) and nuclear factor κB (NF-κB) pathways was inhibited by JZL184 and deletion of MAGL. Further, Brommage et al. investigated the NOTUM lipase in cortical bone and osteoblasts from Notum−/− mice [85]. They reported that inhibition of NOTUM lipase increased cortical bone thickness and strength at multiple skeletal sites in both gonadal intact and ovariectomized rodents. In addition, a study showed a significant reduction in serum activity of the Dpp3 peptidase in OP patients vs. controls and a significant association with bone mass at the femoral neck in patients with severe osteoporosis [86]. In addition, all nitrogen-containing bisphosphonates have been used as first-line drugs for the treatment of osteoporosis, due to their ability to inhibit farnesyl pyrophosphate synthase, an indispensable enzyme for cell function and survival of osteoclasts [87,88].

Oxidative stress results from an imbalance between reactive oxygen species (ROS) production and antioxidant activity. The high concentration of ROS damages cellular membranes, alters the tertiary structure of proteins, and leads to protein degradation. A significant negative correlation in bone tissue has been reported between oxidative stress index and BMD in the lumbar and femoral neck region [89]. These results indicated increased osteoclastic activity and decreased osteoblastic activity. Previous studies reported that estrogen deficiency reduces the defense against oxidative stress in bone and thereby increases skeletal fragility [90,91,92]. The few studies of antioxidant enzyme activity in bone cells suggest that oxidative stress influences osteoblast activity and mineralization [93]. In support of the above evidence, genetic polymorphisms have been associated to oxidative stress and BMD [94].

Recently, the melanosome pathway was found to be involved in bone metabolism. Melanosomes are organelles responsible for the synthesis, storage, and transport of melanin [95,96]. The major components of melanosomes include the tyrosinase enzyme and membrane transport proteins that modulate the melanosomal pH. Briefly, melanosome biogenesis is composed of three stages: the non-pigmented stages of melanosomes (premelanosomes), neutralization of pH (synthesis of melanin), and synthesized melanins fully masked on PMEL fibrils (mature melanosome). In the last stage, mature melanosomes are transferred from melanocytes to keratinocytes and are distributed throughout the skin [95]. In this review, 16 proteins were associated with the melanosome pathway, which could impact bone homeostasis.

The hypothesis suggests that skin pigmentation influences bone maintenance through vitamin D3 concentrations. This includes the fact that humans migrated to higher latitudes in Asia and Europe, with the need for vitamin D3 synthesis as an evolutionary driver for skin lightening [97,98,99]. In line, previous reports have demonstrated variants in genes encoding proteins responsible for the transport, metabolism, and signaling of vitamin D, providing an alternative adaptation mechanism for humans at northern latitudes to avoid vitamin D deficiency [100]. According to the reported proteins, they could participate in protein trafficking and membrane fusion of melanosomes that contribute to skin pigmentation and influence vitamin D synthesis [96]. Nevertheless, many studies aim to understand the relationship between skin pigmentation and loss of bone.

### Challenges in Biomarker Research

**Figure 6:**
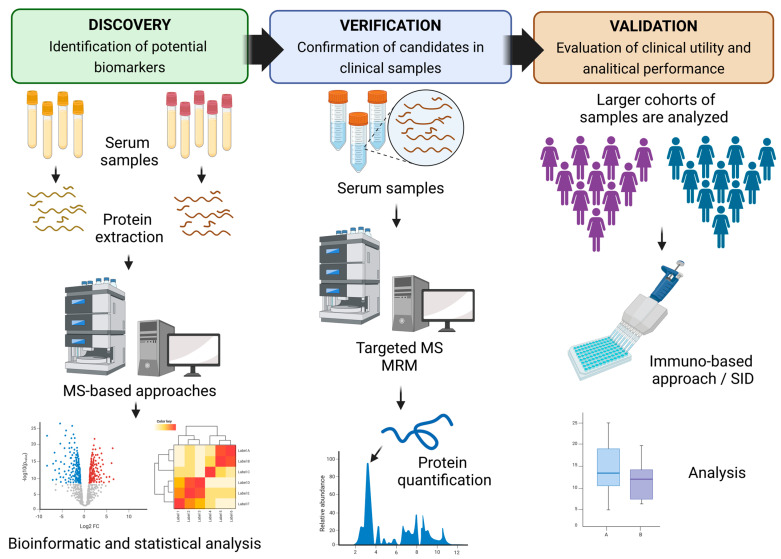
The overall scheme of the pipeline for the development of novel biomarkers. Created with BioRender.com (accessed on 5 July 2024).

Proteomics has increased interest in biomarker research for the prevention and diagnosis of mineral bone loss. In this review, we have introduced bone biomarkers with several proteomic approaches and bone measurement criteria, with a perspective on protein function identification and compressive analysis. Most of the studies were performed following the conventional pipeline for proteomics-based biomarkers. Figure 6 illustrates the overall workflow for the discovery process of a novel proteomic biomarker. The discovery phase involves the criteria selection of individuals recognized as possible covariables and cofounders related to the population. Further, it involves determining the type of biological samples to be analyzed since each sample could require one or more processes. However, during both discovery and targeted proteomic analyses, it is necessary to reduce the complexity of these biological samples due to their high concentrations of albumin and immunoglobulins in order to enhance the detection of lower-abundance proteins. In line, the proteomic approaches also influence the detection of proteins. Subsequently, samples are processed by mass spectrometry (MS), which provides a higher accuracy and sensitivity of quantification of several proteins [101,102]. Due to the high throughput data generated by MS, bioinformatics platforms are required for data screening and analysis. Further, combinate tests are often used to determine the similarity or dissimilarity in composition among samples (e.g., unsupervised or supervised model) [103,104], and statistical tests (e.g., Student’s *t*-test, Wilcoxon test) are incorporated with variables that assess the relationship with disease. Of note is that the number of samples analyzed allows for the reduction in inter-variability among samples and for the determination of biomarkers that may become potential targets for future research. Bioinformatic analysis is performed to identify over- or sub-expressed proteins when compared among conditions.

The verification stage involves targeted proteomics, such as selected reaction monitoring (SRM) and multiple reaction monitoring (MRM) approaches, for promising biomarkers [105] (Figure 6). In this sense, absolute quantitation is achieved by spiking peptides with labeled standards of selected biomarkers. Another technique used is immunohistochemical staining during microscopy, which provides detailed protein localization and the relative abundance of proteins within specific cellular structures. Finally, the validation stage evaluates the biomarker expression between conditions on a large scale [105], usually through an immuno-based approach such as the enzyme-linked immunosorbent assay (ELISA). In addition, the diagnostic value of predictive biomarkers is assessed by receiver operating characteristic (ROC) curves [104]. To summarize, the pipeline and management of each step should be monitored carefully to allow greater throughput, reproducibility, selectivity, and sensitivity for identifying and validating protein biomarkers.

It is crucial to recognize certain limitations inherent to this systematic review that may impact the interpretation of the findings. Firstly, most studies reported in the literature and included in this review were conducted in Asian populations. This issue potentially limits the global translation of the results to other populations, mainly in Admixed or Latin-American populations. Second, genetic variability and environmental factors across diverse ethnic groups could influence proteomic profiles, underscoring the importance of considering population diversity in future investigations. Third, the predominance of case-control designs in the selected studies introduces the possibility of selection bias and precludes the establishment of causal relationships. Another consideration is the heterogeneity in the methodologies used to analyze proteomic samples, ranging from tissue types to quantification techniques. This methodological variability could impact results consistency and comparability across studies. Four, while emphasis has been placed on identifying potential biomarkers, the need for external validation and replication of results on independent cohorts may limit the robustness of conclusions.

## 5. Conclusions

In the broader context of bone health research, the findings of this systematic review contribute significantly to the evolving landscape of biomarker discovery for bone loss. The global burden of osteoporosis and related skeletal disorders underscores the necessity for identifying reliable biomarkers that can aid in early detection and monitoring. Our study primarily focused on proteomic biomarkers associated with bone mineral density, and our results agree with the growing recognition of the multifaceted nature of skeletal health regulation. By synthesizing evidence from diverse studies, our review adds a valuable perspective to the global knowledge of this domain. Identifying potential biomarkers, such as Gelsolin, Annexin A2, and others, provides an establishment for future investigations exploring their clinical applicability in different ethnic groups and geographic regions.

## Figures and Tables

**Table 1:** Studies included in the systematic review.

Author, Year	Country	Ethnicity of Analyzed Population	Study Design	Sample Size (W/M)	Number of Cases	Number of Controls	Mean Age (Years)	Measurement Site (BMD)	Outcome Definition	Confounders
Al-Ansari et al., 2022 [29]	Saudi Arabia	Saudi Arabian	Case-control study	69 (52 W/ 17 M)	47 (OP: 25, OS: 22) 39 W/8 M	22 (13 W/9 M)	Case: (OP: 66.16 ± 1.78; OS: 64.64 ± 1.72) Control: 54.82± 1.03	LS, FN	OS ^a^, OP ^a^	T2DM, thyroid disease, gender, and medication
Chen et al., 2020 [34]	China	Chinese	Case-control study	30 (26 W/4 M)	20 (OP: 10 W/0 M, OS: 9 W/1 M)	10 (7 W/3 M)	Case: (OP: 81 ± 9; OS: 73 ± 11) Control: 76 ± 14	LS, TH	OS ^a^, OP ^a^	Age, BMI, and gender
Daswani et al., 2015 [22]	India	Indian	Case-control study	40 W	10 PEW LBMD (OS: 10) 10 POW LBMD (OS: 10, OP: 9)	20	PEW LBMD: 36.1 ± 1.2 PEW HBMD: 36 ± 1.1, POW LBMD: 55.7 ± 1.1 POW LBMD: 53.8 ± 0.	TH, FN, LS	OS ^a^, OP ^a^	Age, BMI
Deng et al., 2008 [35]	China	Chinese	Case-control study	30 W	15	15	27.3 ± 5.0	TH, FN, (combined value of TR, IR)	BMD ^b^	NR
Deng et al., 2011 [47]	USA	Caucasian	Case-control study	28 W	14	14	LBMD: 67.7 ± 1.7 HBMD: 68.7 ± 1.1	TH	BMD ^c^	Age, gender, height, and weight
Deng et al., 2014 [48]	USA/China	Caucasian	Case-control study	34 W	17	17	LBMD: 50.2 ±1.9 HBMD: 51.8 ± 2.2	TH, FN, (combined value of TR, IR)	BMD ^c^	NR
He et al., 2016 [44]	China	Chinese	Case-control study	20 W	10	10	Case: 56.3 ± 3.61 Normal: 55.0 ± 3.48	LS	OS ^a^	Age, height, and weight
He et al., 2016 [45]	China	Chinese	Case-control study	20 W	10	10	Case: 53.32 ± 2.61 Normal 52.35 ± 1.94	LS	OS ^a^	Age, height, and weight
Huang et al., 2020 [36]	China	Chinese	Case-control study	54 W	OP: 18 OS: 18	18	Case: (OP: 58.33 ± 5.40; OS: 56.72 ± 4.92) Control: 55.22 ± 5.31	LS, TH	OS ^a^, OP ^a^	Age, BMI
Huo et al., 2019 [26]	China	Chinese	Case-control study	84 (61 W/23 M)	OP: 28 (26 W/2 M), OS: 28 (20 W/8 M)	28 (15 W/13 M)	Case: (OP: 73.29 ± 5.25; OS: 67.96 ± 6.28) Control: 68.11 ± 7.56,	NR	OS ^a^, OP ^a^	NR
Li et al., 2023 [37]	China	Chinese	Case-control study	16 W	10	6	Case: 71 ± 1 Control: 65 ± 12	NR	NR	Age, BMI
Martínez-Aguilar et al.,2019 [20]	Mexico	Mexican-Mestizo	Case-control study	30 W	OP: 10, OS: 10,	10	Case: (OP: 75 ± 4; OS: 74 ± 3) Control: 73 ± 2	LS, TH	OS ^a^, OP ^a^	Age, height, weight and BMI
Pepe et al., 2022 [50]	Italy	Italian	Case-control study	24 W	OP: 9, OS: 9	9	Case: (OP: 64.5 ± 9.8; OS: 62.2 ± 7.9) Control: 61.9 ± 6.8	LS, FN	OS ^a^, OP ^a^	Age, BMI
Qundos et al., 2016 [53]	Sweden	Swedish	Case-control study	25 W	16	6	59 to 70	LS, TH	OP ^a^.	NR
Shi et al., 2015 [38]	China	Chinese	Case-control study	25 W	16	9	Case: 61.32 Control: 58	LS	OP ^a^	Age, height, and weight
Shi et al., 2017 [39]	China	Chinese	Case-control study	20 W	10	10	Case: 55.2 ± 2.35 Control: 54.4 ± 2.07	LS	OP ^a^	Age
Xie et al., 2018 [40]	China	Chinese	Case-control study	139 (68 W/71 M)	OP: 31 (23 w/8 m), OS: 46 (21 w/25 m)	26 YN (9 W/17 M) 36 AN (15 W/21 M)	Control: (YN: 34.6 ± 7.4, AN: 64 ± 3.8) Case: (OS: 63 ± 5.3, OP: 63.8 ± 4)	One-third radius site	OS ^a^, OP ^a^	NR
Xu et al., 2020 [41]	China	Chinese	Case-control study	42 (24 W/18 M)	12 W ^S1^/ 9 M ^S2^	12 W ^S1^/ 9 M ^S2^	NR	TH, FN, (combined value of TR, IR)	BMD ^d^	NR
Zeng et al., 2016 [24]	USA	Caucasian	Case-control study	33 W	17	16	LBMD: 50.3 ± 1.86 HBDM: 51.8 ± 2.27	LS, TH (combined value of FN, TR, IR)	BMD ^e^	Age, height, and weight
Zeng et al., 2017 [49]	USA	Caucasian	Case-control study	59 M	29 M	30 M	LBMD: 40.3 ±7.6 HBDM: 41.1 ±7.5	TH, FN, (combined value TR, IR)	BMD ^f^	Age, height, and weight
Zhang et al., 2019 [42]	China	Chinese	Case-control study	30 W	OP: 10, OS: 10	10	63.28 ± 5.78	LS	OS ^a^, OP ^a^	Age
Zhang et al., 2016 [23]	China	Caucasian	Case-control study	42 W	21	21	LBMD: 62.43 ± 9.3 HBDM: 63.95 ± 8.39	LS, TH (combined value of FN, TR, IR)	BMD ^g^	Age, height, and weight
Zhou et al., 2019 [46]	China	Chinese	Case-control study	16 (12 W/4 M)	4 (3 W/1 M)	4 (3 M/1 W)	Case: 56.3 ± 2.3 Control: 54.0 ± 1.1	LS, TH, FN	OP ^a^, non-OP ^h^	Age, BMI
Zhou et al., 2019 [43]	China	Chinese	Case-control study	36 M	LBMD: 9 M OF: 18 M	9 M	OF: 77.3 ± 12.0 LBMD: 70.0 ± 5.4 HBMD: 75.3 ± 7.1	TH (combined value of FN, TR, IR)	OF, BMD ^i^	Age, height, and weight
Zhu et al., 2017 [25]	USA	Caucasian	Case-control study	59 M	29 M	30 M	LBMD: 40.3 ± 7.6 HBDM: 41.1 ± 7.5	TH (combined value of FN, TR, IR)	BMD ^k^	Age, weight, and, height
Nielson et al., 2017 [27]	USA	non-Hispanic white	Cohort	2473 M	accelerated loss *n* = 237 M	BMD maintenance *n* = 453 M	73.6 ± 5.8	TH	BMD ^m^	Age, BMI
Bhattacharyya et al., 2008 [54]	USA	NR	Cross-sectional study	58 W	49 (OP: 28, OS: 21)	8	High turnover group: 80.5 low/normal turnover group: 70.8	LS, TH, mid-distal radius and ulna	Bone turnover	Age, NTX
Grgurevic et al., 2007 [52]	Croatia	Croatian	Cross-sectional study	25 W *	25	-	21 to 60	NR	Acute bone fracture	Age, BMI
Terracciano et al., 2013 [51]	Italy	Italian	Cross-sectional study	61 W	43	18	61.6 ± 9	FN	BMD ^a^	Age, height

Abbreviations: BMD: bone mineral density; OP: osteoporosis; OS: osteopenia; W: women; M: men; NR: not reported; BMI: body mass index; LS = lumbar (L1-L4) spine; TH = total hip; FN = femoral neck; TR: trochanter; IR: intertrochanter; PEW: premenopausal women; POW: postmenopausal women, YN: young normal; AN: aged normal; LBMD: low BMD; HBMD: high BMD; ^a^: BMD WHO criteria (cases: T-score ≤ −2.5 SD; controls: T-score ≥ 1.0 SD); ^b^: HBMD (from top 12%, average Z score ± SD: +1.63 ± 0.16) and LBMD (from bottom 12%, average Z score ± SD: −1.67 ± 0.15) at the hip. ^c^: Z score is defined as the number of standard deviations a subject’s BMD differs from the average BMD of their age-, gender-, and ethnicity-matched populations; ^d^: extremely LBMD (osteopenia/osteoporosis) subjects with LBMD (Z score < −1.30 ± 0.47) and HBMD (Z score > 1.06 ± 0.49); ^e^: HBMD (Z score: 1.32 ± 0.45) and LBMD (Z score: −0.96 ± 0.34); ^f^: LBMD (top 6%) and HBMD (16%) of distribution in Caucasian population; ^g^: HBMD (Z score: 1.02 SD 0.12), LBMD (Z score: –0.76 SD 0.14); ^h^: non-OP (T > − 2.5 at the FN or LS); ^i:^ LBMD (total hip BMD T score: −1.89 ± 0.55) and HBMD (total hip BMDT score: −0.06 ± 0.71). ^k^: LBMD (bottom 30%) and HBMD (top 19%) at the hip, distribution in age- and gender-matched Caucasian population; ^m^: BMD maintenance (no decline; estimated change ≥0 g/cm^2^), expected loss (estimated change between 0 and 1 SD below the estimated mean change, −0.034 g/cm^2^ for FN) and accelerated loss (estimated change ≥1 SD below mean change) and incident hip fracture. * gender distribution not mentioned. Study was designed with two independent samples (S1 and S2).

**Table 2:** Summary of proteomics approaches and the main findings among studies.

Author, Year	Specimen Type	Proteomic Approach	Statistical Analysis/Fold Change Cut-Off	Number of DEPs	Main Findings
Al-Ansari et al., 2022 [29]	Serum	Nano-LC-ESI-MS/MS	ANOVA using post-hoc Tukey’s analysis method, FC >1.5 and <0.67, FDR *p* < 0.05	219	DEPs were associated with humoral immune response, inflammatory response, LXR/RXR activation, FXR/RXR activation, and hematopoiesis. Dysregulation of inflammatory signaling pathways in the LBMD patients.
Chen et al., 2020 [34]	Serum-exosomes	Nano-LC-MS/MS	Mann–Whitney U test *p* < 0.05, FC > 1.2 ^A^	45 ^LH^	Pathways involved with degenerative diseases (Parkinson’s disease and Alzheimer’s disease), and the neuromuscular process of controlling balance.
Daswani et al., 2015 [22]	Peripheral blood monocyte	4—plex iTRAQ LC-MS/MS	Student’s *t*-test *p* < 0.05, FC ≥ 1.5	45 ^LH^	Effect of pHSP27 in monocyte migration towards bone milieu can result in increased osteoclast formation and, thus, contribute to pathogenesis osteoporosis.
Deng et al., 2008 [35]	Peripheral blood monocyte	2DE-MALDI-TOF/TOF	Student’s *t*-test or Kruskal–Wallis test *p* < 0.05, FC ≥ 0.52	38 ^LH^	DEPs might affect CMCs’ trans-endothelium, differentiation, and/or downstream osteoclast functions, thus contribute to differential osteoclastogenesis.
Deng et al., 2011 [47]	Peripheral blood monocyte	LC–nano-ESI-MSE	Kruskal-Wallis Test *p *< 0.05	6 ^LH^	ANXA2 protein significantly promoted monocyte migration across an endothelial barrier in vitro.
Deng et al., 2014 [48]	Peripheral blood monocyte	LC–nano-ESI-MSE	Student’s *t*-test *p* < 0.05	57 ^LH^	Using a proteomics-based multi-disciplinary and integrative study strategy, GSN was significantly down-regulated in premenopausal Caucasians with low vs. high hip BMD.
He et al., 2016 [44]	Serum	WCX-MALDI-TOF-MS	Youden Index, *p* < 0.05	10 ^OSN^	A strategy for screening serum proteins <20 kDa to analyze serum profiles and find potential biomarkers for osteopenia.
He et al., 2016 [45]	Serum	WCX-MALDI-TOF-MS	Youden Index, *p* < 0.05	2 ^OSN^	New serological method for discovering serum protein markers to screen and diagnose osteopenia.
Huang et al., 2020 [36]	Plasma	TMT-LC-MS/MS	Student’s *t*-test, FC > 1.2 and <0.83, *p* < 0.05	208	The differentially abundant proteins exhibited binding, molecular function regulator, transporter and molecular transducer activity, and were involved in metabolic and cellular processes, stimulus response, biological regulation, and immune system processes.
Huo et al., 2019 [26]	Serum-microvesicles	Nano-LC MS/MS	ANOVA using post-hoc Tukey’s analysis method, FC > 2 and <0.5, *p* < 0.05	24 ^LH^	Bone homeostasis-related novel MVs proteins and signaling pathways demonstrated that “integrin signaling pathway” were enriched for osteoporosis. Profilin 1 is verified as a valuable diagnostic indicator for the evaluation of osteoporosis disease.
Li et al., 2023 [37]	Serum	4D-LC-MS/MS	Student’s *t*-test or Mann–Whitney U tests, FC ≥ 2 and ≤0.5, *p* < 0.05	293 ^OPN^	The most significantly enriched GO terms and pathway that the DEPs involved in includes the PI3K–Akt signaling pathway, ECM-receptor interaction, platelet activation, neutrophil extracellular trap formation, as well as complement and coagulation cascades.
Martínez-Aguilar et al., 2019 [20]	Serum	2D DIGE -MALDI TOF/TOF	Student’s *t*-test, FC ≥ 1.5 and ≤1.5, FDR *p* < 0.05	39	VDBP could be considered as a novel biomarker for the early detection of osteoporosis.
Pepe et al., 2022 [50]	Extracellular vesicles blood	Nano-LC-ESI-MS/MS	Unpaired *t*-test or Mann–Whitney U test, *p* ≤ 0.05	140	Bioinformatic analysis revealed the four most represented biological processes, including blood coagulation, gonadropin-releasing hormone receptor, inflammation mediated by chemokine and cytokine signaling, and plasminogen activate cascade pathways.
Qundos et al., 2016 [53]	Plasma	Antibody arrays	Linear model and Wilcoxon rank sum test, *p* < 0.001	7 ^OPN^	AMFR is a potential marker in plasma to differentiate women diagnosed with osteoporosis compared to controls. A decreased gene and protein expression of AMFR may further reflect a lower level of physical activity in osteoporotic patients, when considering that transcripts were abundant in skeletal muscle and mirroring a reduced turnaround in muscle proteins.
Shi et al., 2015 [38]	Serum	MALDI-TOF-MS	Wilcoxon tests using the Youden index, *p* ≤ 0.05	16 ^OPN^	New serological method for the screening and diagnosis of primary type I osteoporosis using serum protein markers.
Shi et al., 2017 [39]	Serum	TMT-LC-ESI-MS/MS	Student’s *t*-test *p* < 0.01, FC ≥ 1.5 and ≤0.67	87 ^OPN^	According to the molecular functions, most of the differentially expressed proteins were involved in binding, catalytic activity and enzyme regulator activity. Candidate biomarkers of postmenopausal osteoporosis were associated with the bone remodeling.
Xie et al., 2018 [40]	Serum exosomes	TMT-LC-MS/MS	One-way ANOVA with a post hoc test *p* < 0.05, FC > 20 and <5	401	Serum-derived exosomes (SDEs) from aged normal volunteers might play a protective role in bone health through facilitating adhesion of bone cells and suppressing aging-associated oxidative stress.
Xu et al., 2020 [41]	Peripheral blood monocyte	LC-MS/MS	Student’s *t*-test *p* < 0.05	331 ^LH^	WNK1, SHTN1, and DPM1 were found differentially expressed between low BMD and high BMD subjects in both genders.
Zeng et al., 2016 [24]	Peripheral blood monocyte	LC-nano-ESI-MS	Student’s *t*-test *p* < 0.05, FC > 1 and <1	30 ^LH^	The contribution of the genes ITGA2B, GSN, and RHOA and the pathways regulation of actin cytoskeleton and leukocyte transendothelial migration to osteoporosis risk.
Zeng et al., 2017 [49]	Peripheral blood monocyte	2D-nano-LC-ESI-MS/MS	Student’s *t*-test *p* < 0.05	35 ^LH^	Numerous pathways/modules including response to elevated platelet cytosolic Ca^2+^, the adherens junction pathway and the leukocyte transendothelial migration pathway, which are thought to be related to osteogenesis, bone formation, and resorption.
Zhang et al., 2019 [42]	Serum	LC-MS/MS	FC > 1.2 and <1/1.2	77 ^OPN^ 77 ^OSN^ 68 ^OPOS^	ApoA-I, Apo A-II, and haptoglobin were mediated with receptors, factors, mechanisms, that related to bone metabolism, while HBD was valuable for diagnosis of osteopenia.
Zhang et al., 2016 [23]	Peripheral blood monocyte	LC-nano-ESI-MSE	Mann–Whitney U test, FC > 1.5 and <1.5	7 ^LH^	Network analysis showed that the module including the annexin gene family was significantly correlated with low BMD, and the lipid-binding and regulating pro-inflammatory cytokines activities were enriched.
Zhou et al., 2019 [46]	Vertebral body-derived bone marrow supernatant fluid	TMT-LC-MS/MS	Wilcoxon-test *p* < 0.05/FC > 1.3	219 ^OPN^	Upregulated proteins were mainly associated with the regulation of transcription and protein metabolism, and downregulated proteins were involved in immune response and movements of the cell and cellular components.
Zhou et al., 2019 [43]	Peripheral blood monocyte	LC-MS/MS	Student’s *t*-test or Unpaired *t*-test with Welch’s correction *p* < 0.05	253 ^OFN^ 13 ^LH^ 8 ^OLH^	ABI1 protein, via promoting osteoblast growth, differentiation and activity, and attenuating monocyte trans-endothelial migration and osteoclast differentiation, influences BMD variation and fracture risk in humans.
Zhu et al., 2017 [25]	Peripheral blood monocyte	LC-nano-ESI-MSE	Student’s *t*-test *p* <0.05	16 ^LH^	ALDOA, MYH14, and Rap1B were identified based on multiple omics evidence, and they may influence the pathogenic mechanisms of osteoporosis by regulating the proliferation, differentiation, and migration of monocytes.
Nielson et al., 2017 [27]	Serum	LC-MS-MS	Markov Chain Monte Carlo meta-fold > 1.1, and meta *p* < 0.1	237 had accelerated hip BMD loss, and 453 maintained hip BMD	CD14 and SHBG were associated with fracture risk; B2MG and TIMP1 have biological role in cellular senescence and aging, and CO7, CO9, CFAD has documented in complement activation and innate immunity functions.
Bhattacharyya et al., 2008 [54]	Serum	LC-MS	Wilcoxon rank-sum test/Student’s *t*-test, FC > 1.5	11	ITIH4 is stored within the bone matrix and is a substrate for enzymatic degradation by osteoclast.
Grgurevic et al., 2007 [52]	Plasma	LC-MS/MS	No comparisons	12	A significant proportion of proteins were of extracellular origin and was involved in the cell growth and proliferation, transport, and coagulation. Several proteins have not been previously identified in the plasma, including: TGF-β-induced protein IG-H3, cartilage acidic protein 1, procollagen C proteinase enhancer protein, and TGF-β receptor III.
Terracciano et al., 2013 [51]	Salivary fluid	MALDI TOF/TOF	NR		α-defensin HNP-1 could be a novel biomarker for osteoporosis.

Abbreviations: MS: mass spectrometer; LC-MS: liquid chromatograph–mass spectrometer; ESI: electrospray ionization; MALDI-TOF-MS: matrix-assisted laser desorption/ionization time-of-flight mass spectrometry; TMT: tandem mass tag; iTRAQ: isobaric tags for relative and absolute quantitation; WCX: weak cationic exchange. A: Data were extracted directly from article original. LH: comparison between low BMD and high BMD; OPN: comparison between osteoporotic patients and normal; OSN: comparison between osteopenic patients and normal; OFN: comparison between patients with osteoporotic fracture and normal. OLH: comparison between patients with osteopenia plus osteoporosis fracture and normal. OPOS: comparison between osteoporotic patients and osteopenic patients.

**Table 3:** Differential expressed proteins reported in at least two studies.

Protein	Sample Type	Direction of Differential Expression in OP/OS/LBMD	Reference
GSN	PBM	↑LBMD	Deng et al., 2008 [35]
	PBM	↓LBMD	Deng et al., 2014 [48]
	PBM	↓LBMD	Zeng et al., 2016 [24]
	Serum	↓OP/OS	Martínez-Aguilar et al., 2019 [20]
ANXA2	PBM	↑LBMD	Deng et al., 2011 [47]
	PBM	↓LBMD	Daswani et al., 2015 [22]
	PBM	↑LBMD	Zhang et al., 2016 [23]
APOA1	EVB	↑OS/OP	Pepe et al., 2022 [50]
	Serum	↓OS—↑OP	Zhang et al., 2016 [23]
PPIA	PBM	↓LBMD	Deng et al., 2011 [47]
	PBM	↓LBMD	Zhang et al., 2016 [23]
P4HB	PBM	↓LBMD	Deng et al., 2008 [35]
	PBM ^M^	↓LBMD	Zeng et al., 2017 [49]
ITGB1	Serum exosomes ^WM^	↓OP	Zeng et al., 2017 [49]
	PBM ^M^	↑LBMD	Xie et al., 2018 [40]
ITGA2B	PBM	↑LBMD	Deng et al., 2014 [48]
	PBM	↓LBMD	Zeng et al., 2016 [24]
MYH14	PBM ^M^	↑LBMD	Zhu et al., 2017 [25]
	Serum ^WM^	↓OP	Al-Ansari et al., 2022 [29]
VWF	EVB	↓OS/OP	Pepe et al., 2022 [50]
	Serum	↑OP	Li et al., 2023 [37]
LOC654188	PBM	↓LBMD	Deng et al., 2011 [47]
	PBM	↓LBMD	Zhang et al., 2016 [23]

Abbreviations: ↓ downregulation; ↑ upregulation; PBM: peripheral blood monocytes; EVB: extra-. cellular vesicles; LBMD: low bone mineral density; OP: osteoporosis; OS: osteopenia. M: men; WM: women and men.

## Data Availability

The datasets analyzed in this study are available in the Supplementary Material and could be available from the corresponding author.

## Supplementary Materials

The following supporting information can be downloaded at: https://www.mdpi.com/article/10.3390/ijms25147526/s1.
