# Supplementary material for: Proteomic Biomarkers Associated with Low Bone Mineral Density: A Systematic Review
Source: Int J Mol Sci. 2024 Jul 9;25(14):7526. doi: 10.3390/ijms25147526 (PMC11277462; doi:10.3390/ijms25147526)
Supplement: Supplementary file 1 [file ijms-25-07526-s001.zip › ijms-3089019-supplementary.pdf]

Table S1. Summary of proteomics approaches and differentially expressed proteins among studies.

Author, Year	Specimen type	Proteomic approach	Relevant differentially expressed proteins
Al-Ansari et al., 2022 [18]	Serum	Nano-LC-ESI-MS/MS	Immunoglobulin Heavy Constant Gamma 2 (IGHG2), Complement C3 (C3), RNA binding protein (MEX3B), C-Reactive Protein (CRP), immunoglobulin Lambda constant 1 (IGLC1), myosin heavy chain 14 (MYH14), Complement C1q C Chain (C1QC)
Chen et al., 2020 [23]	Serum-exosomes	Nano-LC-MS/MS	Proteasome subunit beta type-9 (PSMB9), Alanine–tRNA ligase, cytoplasmic (AARS), Poly(rC)-binding protein 2 (PCBP2), and V-type immunoglobulin domain-containing suppressor of T-cell activation (VSIR)
Daswani et al., 2015 [45]	Peripheral blood monocyte	4 - plex iTRAQ LC-MS/MS	Heatshock protein 27 (HSPB1 or HSP27), Mitochondrial Rho GTPase 2 (RHOT2), Protein YIF1B (YIF1B), ATP-binding cassette sub-family B member 6 (ABCB6), Neutrophil defensin 1 (DEFA1), Sulfotransferase 1A1(SULT1A1), Ras-related C3 botulinum toxin substrate 1 (RAC1), RMCX3, Small ribosomal subunit related-proteins (RPS12 , RPS28), Protein arginine N-methyltransferase 1 (PRMT1), DNA cross-link repair 1A protein (DCLRE1A), Vacuolar protein sorting-associated protein 37A (VPS37A), Solute carrier family 2 facilitated glucose transporter member 3 (SLC2A3), Transmembrane emp24 domain-containing protein 3 (TMED3), T-complex protein 1 subunit delta (CCT4), Annexin-related proteins (ANXA1, ANXA2, ANXA6), Lysozyme C (LYZ), Tubulin beta-6 chain (TUBB6), Histone-related proteins (HIST1H4I, HIST1H1B, HIST1H2BD, HIST1H2BB), Protein SET (SET), Heterogeneous nuclear ribonucleoprotein-related proteins (HNRNPU, HNRNPUL2, HNRNPD, HNRNPC), X-ray repair cross-complementing protein 5 (XRCC5), Vimentin (VIM), Myeloid cell nuclear differentiation antigen (MNDA), Non-secretory ribonuclease (RNASE2), ER lumen protein-retaining receptor 1 (KDELR1), Signal transduction-associated protein 1 (KHDRBS1), Apoptosis-associated speck-like protein containing a CARD (PYCARD), Deoxynucleoside triphosphate triphosphohydrolase (SAMHD1), HLA class II histocompatibility antigen (HLA-DRA), Calcium-regulated heat-stable protein 1 (CARHSP1), Macrophage-capping protein (CAPG), Formin-like protein 1 (FMNL1), SPRY domain-containing protein 4 (SPRYD4), Serine/threonine-protein phosphatase (CPPED1), Elongation factor 1-delta (EEF1D)

Deng et al., 2008 [24]	Peripheral blood monocyte	2DE-MALDI-TOF/TOF	Ras suppressor protein1 (RSU1), gelsolin (GSN), manganese containing superoxide dismutase (SOD2), glutathione peroxidase 1 (GPX1), and prolyl 4 hydroxylase b subunit (P4HB)
Deng et al., 2011 [37]	Peripheral blood monocyte	LC–nano-ESI-MSE	Annexin A2 (ANXA2; IPI00418169.3, IPI00455315.4, and IPI00797556.1), Peptidyl-prolyl cis-trans isomerase A (PPIA; IPI00419585.9), similar to peptidylprolyl isomerase A-like (PPIAP19; IPI00374732.4), similar to peptidyl-prolyl isomerase A-like (LOC654188; IPI00887678.1)
Deng et al., 2014 [38]	Peripheral blood monocyte	LC–nano-ESI-MSE	Gelsolin (GSN, IPI00641047.5), Ras-Related Protein Rab-27B (RAB27B, IPI00010491.3), Ras-Related Protein Rap-1b (RAP1B, IPI00816182.1), Stomatin (STOM, IPI00219682.6), Integrin Subunit Alpha 2b (ITGA2B, IPI00385519.1, IPI00747241.1), Glyceraldehyde-3-Phosphate Dehydrogenase (GAPDH, IPI00794605.1)
He et al., 2016 [34]	Serum	WCX-MALDI-TOF-MS	Secretin (SCT)
He et al., 2016 [35]	Serum	WCX-MALDI-TOF-MS	Protein kish-B (KISHB) and C-X-C motif chemokine 6 (CXCL6)
Huang et al., 2020 [25]	Plasma	TMT-LC-MS/MS	Lysozyme C (LYZ), Glucosidase alpha neutral AB (GANAB), Protein disulfide isomerase A5 (PDIA5), Complement C4-A (C4A), Uncharacterized protein DKFZp666N164 (DKFZp666N164), Adipocyte plasma membrane-associated protein (APMAP), cDNA FLJ51711 (B4DE30)
Huo et al., 2019 [26]	Serum-microvesicles	Nano-LC MS/MS	Vinculin (VCL), Filamin A (FLNA), Profilin 1 (PFN1)

Li et al., 2023 [27]	Serum	4D-LC-MS/MS	Cadherin 1 (CDH1), Insulin Like Growth Factor Binding Protein 2 (IGFBP2), Von Willebrand Factor (VWF), FAM3 Metabolism Regulating Signaling Molecule C (FAM3C), Peroxiredoxin 6 (PRDX6), and Purine Nucleoside Phosphorylase (PNP)
Martínez-Aguilar et al., 2019 [49]	Serum	2D DIGE - MALDI TOF/TOF	Vitamin D Receptor (VDBP), Ceruloplasmin (CP), and Gelsolin (GSN)
Pepe et al., 2022 [43]	Extracellular vesicles blood	Nano-LC-ESI-MS/MS	Von Willebrand factor (VWF), apolipoprotein AI (APOA1), myosin reactive immunoglobulin light chain variable region (IGVL), immunoglobulin mu chain C region, IG γ chain C region, fibrinogen α chain (FGA), fibrinogen β chain (FGB), fibrinogen γ chain (FGG), IG kappa variable 2D2 (IGKV2D), carboxyl ester hydrolase protein (B7Z795)
Qundos et al., 2016 [48]	Plasma	Antibody arrays	Autocrine Motility Factor Receptor (AMFR), progestagen-associated endometrial protein (PAEP), and homeobox protein notochord (NOTO)
Shi et al., 2014 [28]	Serum	MALDI-TOF-MS	C-C motif of chemokine 23 (CCL23); C-C motif of chemokine 2 (LECT2); 13,745.480 Da matched leukocyte cell-derived chemotaxin 1 (CNMD); and 15,114.52 Da matched gamma-parvin (PARVG)
Shi et al.,2017 [29]	Serum	TMT-LC-ESI-MS/MS	Ras-related protein Rab-7a (RAB7A), Thrombospondin-1 (TSP1), Growth arrest-specific protein 6 (GAS6), Secreted phosphoprotein 24 (SPP24)

Xie et al., 2018 [30]	Serum -Exosomes	TMT-LC-MS/MS	Integrin b1 (ITGB1), integrin b3 (ITGB3), Transforming Growth Factor Beta 1 (TGFb1), and Amyloid Beta Precursor Protein (APP)
Xu et al., 2021 [31]	Peripheral blood monocyte	LC-MS/MS	WNK Lysine Deficient Protein Kinase 1 (WNK1), Shootin1 (SHTN1), Dolichyl-Phosphate Mannosyl-transferase Subunit 1 (DPM1).
Zeng et al., 2016 [40]	Peripheral blood monocyte	LC-nano-ESI-MS	Integrin alpha 2b (ITGA2B), Gelsolin (GSN)
Zeng et al., 2017 [39]	Peripheral blood monocyte	2D-nano-LC-ESI-MS/MS	Thrombospondin Receptor (CD36), Actinin Alpha 1 (ACTN1), Prolyl 4-Hydroxylase Subunit Beta (P4HB), Integrin Subunit Beta 1 (ITGB1)
Zhang et al., 2019 [32]	Serum	LC-MS/MS	Apolipoprotein A-I (APOA1), apolipoprotein A-II (APOA2), hemoglobin subunit alpha (HBG2), and haptoglobin (HP)
Zhang et al., 2016 [41]	Peripheral blood monocyte	LC-nano-ESI-MS[E]	Similar to peptidyl-prolyl<br><br>isomerase A-like (LOC654188), Peptidyl-prolyl cis-trans isomerase A (PPIA), Transgelin-2 (TAGLN2), and 14-3-3 protein beta/alpha (YWHAB) -BMD / Lamin-B1 (LMNB1), Putative annexin A2-like protein (ANXA2P2), and PPIA (ANXA2)-BMD
Zhou et al., 2019 [36]	Vertebral body-derived bone marrow supernatant fluid	TMT-LC-MS/MS	Probable ATP-dependent RNA helicase DDX5 (DDX5), 26S proteasome regulatory subunit 7 (PSMC2), Casein kinase I isoform alpha (CSNK1A1), Perilipin-1 (PLIN1), Integrin-linked protein kinase (ILK), and Tropomyosin alpha-4 chain (TPM4)

Zhou et al., 2019 [33]	Peripheral blood monocyte	LC-MS/MS	Cytochrome b-c1 complex subunit 2 mitochondrial (QCR2), ATP synthase subunit delta, mitochondrial (ATPD), Trifunctional enzyme subunit alpha, mitochondrial (ECHA), Torsin-4A (TOR4A), Gamma-interferon-inducible lysosomal thiol reductase (GILT), DNA primase large subunit (PRI2), Protein S100-A9 (S100A9), Abl Interactor 1(ABI1)
Zhu et al., 2016 [42]	Peripheral blood monocyte	LC-nano-ESI-MSE	Aldolase, fructose-bisphosphate A (ALDOA), Myosin heavy chain 14 (MYH14), and Member of RAS oncogene family (Rap1B)
Nielson et al., 2017 [47]	Serum	LC-MS-MS	Monocyte differentiation antigen (CD14), Neural cell adhesion molecule, Complement component C7 (C7), IgGFc-binding protein (FCGBP),Alpha-2-macroglobulin (A2M), L1-like protein (CHL1), Complement factor D (CFD), Immunoglobulin kappa constant (IGKC), Lectin, galactoside-binding soluble 3 binding protein (LGALS3BP), Sex hormone binding globulin (SHBG)
Bhattacharyya et al., 2008 [50]	Serum	LC-MS	Interalpha-trypsin-inhibitor<br><br>heavy chain H4 precursor (ITIH4)
Grgurevic et al., 2007 [46]	Plasma	LC-MS/MS	Unclear mentioned: cartilage acidic protein 1 (CRTAC-1), metalloproteinase<br><br>inhibitor 1 (TIMP-1), TGF-β receptor III (TGFBR3), Splice isoform A of the proteoglycan-4 (like PRG4)
Terracciano et al., 2013 [44]	Salivary fluid	MALDI TOF/TOF	α- defensin HNP-1

Abbreviations: MS: mass spectrometer ; LC-MS: liquid chromatograph–mass spectrometer; HPLC: high-resolution liquid chromatography; ESI: Electrospray Ionization; MALDI-TOF-MS: matrix-assisted laser desorption/ionization time-of-flight mass spectrometry; TMT: Tandem Mass Tag; iTRAQ: isobaric tags for relative and absolute quantitation; UPLC: NanoAcquity Ultra Performance Liquid Chromatography; HDMS: Synapt High Definition Mass

Spectrometry; WCX: weak cationic exchange. A: Data were extracted directly from article original. LH: Comparison between low-BMD and high-BMD; OPN: Comparison between osteoporotic patients and normal; OSN: Comparison between osteopenic patients and normal; OFN: Comparison between patients with osteoporotic fracture and normal. OLH: Comparison between patients with osteopenia plus osteoporosis fracture and normal.

Table S2. Characteristics of subjects and proteins validated among selected studies.

Author, Year	Technique validation	Sample size (W/M)	Number of cases	Number of controls	Mean age (years)	Validated proteins
Al-Ansari et al., 2022 [18]	MRM	-	-	-	-	IGHG2, C3, MEX3B, CRP, IGLC1, MYH14, and C1QC
Chen et al., 2020 [23]	PRM	30	OP: 20	10	-	PSMB9, AARS, PCBP2, and VSIR
Daswani et al., 2015 [45]	ELISA	40	20	20	PEW LBMD: 35.7 ± 0.8; HBMD: 34.8 ± 1.2 / POW LBMD: 54.2 ± 0.9; HBMD: 53.6 ± 0.9.	HSP27
Deng et al., 2008 [24]	WB	-	-	-	-	Ras suppressor protein1 (RSU1), Gelsolin (GSN), manganese containing superoxide dismutase (SOD2), glutathione peroxidase 1(GPX1), and prolyl 4-hydroxylase b subunit (P4HB)
Deng et al., 2011 [37]	WB Multiomics analysis (DNA, RNA)	80	LBMD: 40	HBMD: 40	LBMD: 50.0 ± 1.3<br>HBMD: 49.4 ± 1.3	ANXA2 protein (IPI00418169.3, IPI00455315.4, and IPI00797556.1)
Deng et al., 2014 [38]	WB	Cohort 2:29<br>Cohort 3:40	Cohort 2 LBMD: 14<br>Cohort 3 LBMD:20	Cohort 2 HBMD:15<br>Cohort 3 HBMD:20	Cohort 2 HBMD: 51.0 ± 1.8 - LBMD: 50.1 ± 2.1 / Cohort 3 HBMD: 41.7 ± 1.8 - LBMD: 42.3 ± 1.8	Gelsolin (GSN)
He et al., 2016 [34]	ELISA	80	40	40	OS: 56.56 ± 3.78<br>Control: 56.10 ± 3.50	Secretin
Huang et al., 2020 [25]	PRM	-	-	-	-	Lysozyme C, Glucosidase and Protein disulfide isomerase A5
Huo et al., 2019 [26]	WB, ELISA	18^{SN}	12<br>(OP: 6, OS: 6)	6	NR	Profilin 1, Filamin A, and Vinculin

Li et al., 2023 [27]	ELISA	72	36	36	OP: 71 ± 1<br>Control: 65 ± 12	CDH1, IGFBP2, VWF, and PNP
Martínez-Aguilar et al., 2019 [49]	ELISA	74	48<br>(OP: 19, OS: 29)	26	OP: 73±9; OS: 67± 7<br>Control: 65±8	VDBP
Qundos et al., 2016 [48]	WB	180	Set 1: 46<br>Set 2:45	Set 1: 44<br>Set 2:45	Set 1 Cases: 65, Control: 63 / Set 2 Cases:73, Control: 72	AMFR
Xie et al., 2018 [30]	ELISA	72[SN]	37<br>(OP:18, OS:19)	35	NR	ITGB1,<br>ITGB3
Zhou et al., 2019 [36]	WB	16<br>(12W/4M)	8<br>(6W/2M)	8<br>(6W/2M)	Cases: OP 57.1 ± 1.4<br>Normal: 56.1 ± 1.5	DDX5, PSMC2, PLIN1, ILK, and TPM4
Zhou et al., 2019 [33]	WB, ELISA	159M	90M<br>(sample 3 LBMD 32, OF: 20 / sample 4 LBMD:38)	69M<br>(sample 3 HBMD: 32 / sample 4 HBMD :37)	Sample 3 LBMD: 70.0 ± 3.3, OF: 72.0 ± 13.4 /<br>Sample 4 LBMD: 70.2 ± 2.7	ABI1

Abbreviations: W: Women; M: Male; OP: osteoporosis; OS: osteopenia; LBMD: Low bone mineral density; HBMD: High bone mineral density; OF: patients with osteoporotic fracture; MRM: Multiple reaction monitoring; PRM: parallel reaction monitoring analysis; WB: Western blot; GA: Genetic algorithm; SN: sex not reported.

Table S3. The Joanna Briggs Institute Critical Appraisal Checklist for analytical case-control studies.

Study (Ref)	1. Were the groups comparable other than the presence of disease in cases or the absence of disease in controls?	2. Were cases and controls matched appropriately?	3. Were the same criteria used for identification of cases and controls?	4. Was exposure measured in a standard, valid and reliable way?	5. Was exposure measured in the same way for cases and controls?	6. Were confounding factors identified?	7. Were strategies to deal with confounding factors stated?	8. Were outcomes assessed in a standard, valid and reliable way for cases and controls?	9. Was the exposure period of interest long enough to be meaningful?	10. Was appropriate statistical analysis used?	Risk of bias
Al-Ansari et al., 2022	Yes	Yes	Yes	Yes	Yes	Yes	Yes	Yes	Not applicable	Yes	Low
Chen, M., Li, 2020	Yes	No	Yes	Yes	Yes	Yes	Yes	Yes	Not applicable	Yes	Low
Daswani, B., 2015	Yes	Yes	Yes	Yes	Yes	Yes	Yes	Yes	Not applicable	Yes	Low
Deng, F. , 2011	Yes	Yes	Not applicable	Yes	Yes	No	No	Yes	Not applicable	Yes	Low
Deng, F. , 2008	Yes	Yes	Yes	Yes	Yes	No	No	Yes	Not applicable	Yes	Low
Deng, F. ,2014	Yes	Not applicable	Not applicable	Yes	Yes	No	No	Yes	Not applicable	Yes	Low
He, W. T., Liang, B. C., 2016	Yes	Yes	Yes	Yes	Yes	Yes	Yes	Yes	Not applicable	Yes	Low
He, W. T., Liang, B. C., 2016	Yes	Yes	Yes	Yes	Yes	Yes	Yes	Yes	Not applicable	Yes	Low
Huang, D., 2020	Yes	Yes	Yes	Yes	Yes	Yes	Yes	Yes	Not applicable	Yes	Low
Huo C, 2019	Yes	Yes	Yes	Yes	Yes	No	No	Yes	Not applicable	Yes	Low
Li, C., 2023	Yes	Yes	Yes	Yes	Yes	Yes	Yes	Yes	Not applicable	Yes	Low
Martínez-Aguilar,2019	Yes	Yes	Yes	Yes	Yes	Yes	Yes	Yes	Not applicable	Yes	Low

Pepe, J.,2022	Yes	Yes	Yes	Yes	Yes	Yes	Yes	Yes	Not applicable	Yes	Low
Qundos, U.,2016	Yes	No	Yes	Yes	Yes	No	No	Yes	Not applicable	Yes	Low
Shi, X. L.,2014	Yes	Yes	Yes	Yes	Yes	Yes	Yes	Yes	Not applicable	Yes	Low
Shi, X. L.,2017	Yes	Yes	Yes	Yes	Yes	Yes	Yes	Yes	Not applicable	Yes	Low
Xie, Y.,2018	Yes	No	Yes	Yes	Yes	No	No	Yes	Not applicable	Yes	Low
Xu, Q.,2021	Yes	Yes	Yes	Yes	Yes	Yes	Yes	Yes	Not applicable	Yes	Low
Zeng, Y., 2016	Yes	Yes	Yes	Yes	Yes	Yes	Yes	Yes	Not applicable	Yes	Low
Zeng, Y., 2017	Yes	Yes	Yes	Yes	Yes	Yes	Yes	Yes	Not applicable	Yes	Low
Zhang LL, 2019	Yes	Yes	Yes	Yes	Yes	Yes	No	Yes	Not applicable	Yes	Low
Zhang, L.,2016	Yes	Yes	Yes	Yes	Yes	Yes	Unclear	Yes	Not applicable	Yes	Low
Zhou, Q., Xie, F., 2019	Yes	Yes	Yes	Yes	Yes	Yes	Yes	Yes	Not applicable	Yes	Low
Zhou, X., Li, C. H.,2019	Yes	Yes	Yes	Yes	Yes	Yes	Yes	Yes	Not applicable	Yes	Low
Zhu, W., 2017	Yes	Yes	Yes	Yes	Yes	Yes	Yes	Yes	Not applicable	Yes	Low

Table S4. The Joanna Briggs Institute Critical Appraisal Checklist for analytical cross-sectional studies.

Study (Ref)	1. Were the criteria for inclusion in the sample clearly defined?	2. Were the study subjects and the setting described in detail?	3. Was the exposure measured in a valid and reliable way?	4. Were objective, standard criteria used for measurement of the condition?	5. Were confounding factors identified?	6. Were strategies to deal with confounding factors stated?	7. Were the outcomes measured in a valid and reliable way?	8. Was appropriate statistical analysis used?	Risk of bias
Bhattacharyya, 2008	Yes	Yes	Not applicable	Yes	Yes	Yes	Yes	Yes	Low
Grgurevic, L., 2007	No	No	Not applicable	Yes	Yes	Yes	Yes	Yes	Low
Terracciano, R.,2013	Yes	Yes	Not applicable	Yes	Yes	Yes	Yes	Yes	Low

Table S5. The Joanna Briggs Institute Critical Appraisal Checklist for analytical cohort studies.

Study (Ref)	1. Were the two groups similar and recruited from the same population?	2. Were the exposures measured similarly to assign people to both exposed and unexposed groups?	3. Was the exposure measured in a valid and reliable way?	4. Were confounding factors identified?	5. Were strategies to deal with confounding factors stated?	6. Were the groups/participants free of the outcome at the start of the study (or at the moment of exposure)?	7. Were the outcomes measured in a valid and reliable way?	8. Was the follow up time reported and sufficient to be long enough for outcomes to occur?	9. Was follow up complete, and if Not, were the reasons to loss to follow up described and explored?	10. Were strategies to address incomplete follow up utilized?	11. Was appropriate statistical analysis used?	Risk of bias
Nielson, C. M., 2017	Yes	Yes	Yes	Yes	Yes	Yes	Yes	Yes	Yes	Not applicable	Yes	Low

Table S6. Pathways associated to differentially expressed proteins among studies.

GO-term or pathway	Description	Matching proteins in network
BTO:0001486	Skeletal system	A2M, ANXA1, **ANXA2**, APMAP, **APOA1**, APOA2, APP, C3, C4A, CAPG, CD14, CD36, CP, CRP, CRTAC1, DCLRE1A, DEFA1, EEF1D, FAM3C, FCGBP, FGA, FGB, FGG, FMNL1, GANAB, GAPDH, HNRNPC, HSPB1, IGFBP2, **ITGA2B**, **ITGB1**, ITGB3, LGALS3BP, PRDX6, SLC2A3, SPP2, STOM, TAGLN2, TGFB1, THBS1, TIMP1, VIM, **VWF**, XRCC5
GO: 0002376 / GO: 0051246	Immune system process / Regulation of protein metabolic process	A2M, AARS1, ABI1, **ANXA2**, **APOA1**, APP, C3, C4A, CD36, CDH1, CSNK1A1, EEF1D, FGA, FLNA, GAPDH, GAS6, GSN, H1-5, HNRNPD, HNRNPU, HSPB1, ILK, ITGB3, KHDRBS1, PAEP, **PPIA**, PRMT1, PSMB9, PSMC2, PYCARD, RAB7A, RAC1, S100A9, SET, SPP2, TGFB1, THBS1, TIMP1, VIM, VSIR, WNK1, XRCC5, YWHAB
GO: 0023051	Regulation of signaling	AMFR, ANXA1, **ANXA2**, **APOA1**, APP, C3, CCL23, CD14, CD36, CDH1, CSNK1A1, DDX5, EEF1D, FGA, FGB, FGG, FLNA, GAPDH, GAS6, HSPB1, IGFBP2, ILK, **ITGB1**, ITGB3, **P4HB**, PPIA, PRMT1, PYCARD, RAB7A, RAC1, RAP1B, RPS12, S100A9, SAMHD1, SCT, SOD2, TGFB1, TGFBR3, THBS1, TIMP1, VWF, WNK1, YWHAB
GO:0006810	Transport	ABCB6, ANXA1, **ANXA2**, ANXA6, **APOA1**, APOA2, APP, ATP5F1D, CCT4, CD14, CD36, CP, FGG, FLNA, GAS6, GC, **GSN**, HNRNPU, HSPB1, **ITGB1**, ITGB3, KDELR1, LGALS3BP, LMNB1, MRS2, PYCARD, RAB27B, RAB7A, RAC1, RAP1B, RHOT2, SCN2A, SCT, SLC2A3, TGFB1, THBS1, TMED3, TMEM167B, VPS37A, WNK1, YIF1B, YWHAB
GO: 0022607	Cellular component assembly	ABCB6, ACTN1, ALDOA, **ANXA2**, **APOA1**, APOA2, APP, ATP5F1D, CAPG, CDH1, FGA, FGB, FGG, FLNA, GAS6, **GSN**, H1-5, H2BC3, H2BC5, H4C6, HLA-DRA, **ITGB1**, ITGB3, LMNB1, NOTO, PARVG, PRMT1, PYCARD, RAB7A, RAC1, RPS28, SAMHD1, SET, SHTN1, SOD2, TGFB1, TGFBR3, VCL, VPS37A, XRCC5
GO: 0030154	Cell differentiation	A2M, ABI1, ACTN1, ANXA1, **ANXA2**, ANXA6, APP, C3, CDH1, CHL1, CNMD, CRTAC1, DDX5, FGG, FLNA, GAS6, HNRNPC, HNRNPU, ILK, **ITGB1**, ITGB3, KDELR1, NOTO, PARVG, **PPIA**, PRMT1, SMC2, RAC1, RAP1B, S100A9, SHTN1, SOD2, TAGLN2, TGFB1, TGFBR3, TPM4, VCL, VIM, WNK1, XRCC5
HSA-109582	Hemostasis	A2M, ACTN1, ALDOA, **ANXA2**, APOA1, APP, CD36, FAM3C, FGA, FGB, FGG, FLNA, GAS6, **ITGA2B**, **ITGB1**, ITGB3, LGALS3BP, PFN1, PPIA, RAB27B, RAC1, RAP1B, SPP2, TAGLN2, TGFB1, THBS1, TIMP1, TOR4A, TUBB6, VCL, **VWF**
HSA-1474244	Extracellular matrix organization	A2M, ACTN1, APP, CDH1, FGA, FGB, FGG, **ITGA2B**, **ITGB1**, ITGB3, **P4HB**, TGFB1, THBS1, TIMP1, **VWF**

The proteins find in at least two studies have been highlighted in bold face.

Table S7. ClueGo pathways associated to protein discovery among studies.

ID	Term	Ontology Source	Term PValue	Term PValue Corrected with Bonferroni step down	Group PValue	Group PValue Corrected with Bonferroni step down	GOLevels	GOGroups	% Associated Genes	Nr. Genes	Associated Genes Found
GO:0030141	secretory granule	GO_CellularComponent-EBI-UniProt-GOA-ACAP-ARAP_25.05.2022_00h00	0.00	0.00	0.00	0.00	[3, 5, 6, 8]	Group09	4.29	41.00	[A2M, ACTN1, ALDOA, ANXA2, APOA1, APP, C3, CD14, CD36, CPPED1, DEFA1, FAM3C, FGA, FGB, FGG, GAS6, GSN, ITGA2B, ITGB3, LGALS3BP, LYZ, MNDA, PNP, PPIA, PRDX6, PSMC2, PYCARD, RAB27B, RAB7A, RAC1, RNASE2, S100A9, SLC2A3, SPP2, STOM, THBS1, TIMP1, TOR4A, VCL, VWF, XRCC5]
GO:0034774	secretory granule lumen	GO_CellularComponent-EBI-UniProt-GOA-ACAP-ARAP_25.05.2022_00h00	0.00	0.00	0.00	0.00	[4, 5, 6, 7, 8, 9	Group09	8.82	32.00	[A2M, ACTN1, ALDOA, ANXA2, APOA1, APP, C3, CPPED1, DEFA1, FAM3C, FGA, FGB, FGG, GAS6, GSN, LGALS3BP, LYZ, MNDA, PNP, PPIA, PRDX6, PSMC2, PYCARD, RNASE2, S100A9, SPP2, THBS1, TIMP1, TOR4A, VCL, VWF, XRCC5]
GO:0034774	secretory granule lumen	GO_CellularComponent-EBI-UniProt-GOA-ACAP-ARAP_25.05.2022_00h00	0.00	0.00	0.00	0.00	[4, 5, 6, 7, 8, 9	Group20	8.82	32.00	[A2M, ACTN1, ALDOA, ANXA2, APOA1, APP, C3, CPPED1, DEFA1, FAM3C, FGA, FGB, FGG, GAS6, GSN, LGALS3BP, LYZ, MNDA, PNP, PPIA, PRDX6, PSMC2, PYCARD, RNASE2, S100A9, SPP2, THBS1, TIMP1, TOR4A, VCL, VWF, XRCC5]
GO:0050839	cell adhesion molecule binding	GO_MolecularFunction-EBI-UniProt-GOA-ACAP-ARAP_25.05.2022_00h00	0.00	0.00	0.00	0.00	[3]	Group15	4.18	25.00	[ABI1, ACTN1, ALDOA, ANXA1, ANXA2, CAPG, CDH1, EEF1D, FGA, FGB, FGG, FLNA, ITGA2B, ITGB1, ITGB3, P4HB, PFN1, PPIA, PRDX6, SHTN1, TAGLN2, THBS1, VCL, VWF, YWHAB]
GO:0050839	cell adhesion molecule binding	GO_MolecularFunction-EBI-UniProt-GOA-ACAP-ARAP_25.05.2022_00h00	0.00	0.00	0.00	0.00	[3]	Group20	4.18	25.00	[ABI1, ACTN1, ALDOA, ANXA1, ANXA2, CAPG, CDH1, EEF1D, FGA, FGB, FGG, FLNA, ITGA2B, ITGB1, ITGB3, P4HB, PFN1, PPIA, PRDX6, SHTN1, TAGLN2, THBS1, VCL, VWF, YWHAB]
GO:0005925	focal adhesion	GO_CellularComponent-EBI-UniProt-GOA-ACAP-ARAP_25.05.2022_00h00	0.00	0.00	0.00	0.00	[5]	Group20	4.59	21.00	[ACTN1, ANXA1, ANXA6, FLNA, GSN, HSPB1, ILK, ITGA2B, ITGB1, ITGB3, P4HB, PARVG, PCBP2, PFN1, PPIA, RAC1, RSU1, TPM4, VCL, VIM, YWHAB]
GO:0042060	wound healing	GO_BiologicalProcess-EBI-UniProt-GOA-ACAP-ARAP_25.05.2022_00h00	0.00	0.00	0.00	0.00	[4]	Group15	4.25	20.00	[ACTN1, ANXA1, ANXA2, ANXA6, CD36, FGA, FGB, FGG, FLNA, GAS6, GPX1, HSPB1, ILK, ITGB1, ITGB3, PPIA, THBS1, TIMP1, VCL, VWF]
GO:0006909	phagocytosis	GO_BiologicalProcess-EBI-UniProt-GOA-ACAP-ARAP_25.05.2022_00h00	0.00	0.00	0.00	0.00	[5]	Group19	5.68	20.00	[ANXA1, APOA1, APOA2, C3, C4A, CD14, CD36, CRP, GAS6, GSN, HNRNPC, IGHG2, IGKC, IGLC1, ITGB1, ITGB3, PYCARD, RAB7A, RAC1, THBS1]
GO:0042060	wound healing	GO_BiologicalProcess-EBI-UniProt-GOA-ACAP-ARAP_25.05.2022_00h00	0.00	0.00	0.00	0.00	[4]	Group20	4.25	20.00	[ACTN1, ANXA1, ANXA2, ANXA6, CD36, FGA, FGB, FGG, FLNA, GAS6, GPX1, HSPB1, ILK, ITGB1, ITGB3, PPIA, THBS1, TIMP1, VCL, VWF]
GO:0006909	phagocytosis	GO_BiologicalProcess-EBI-UniProt-GOA-ACAP-ARAP_25.05.2022_00h00	0.00	0.00	0.00	0.00	[5]	Group21	5.68	20.00	[ANXA1, APOA1, APOA2, C3, C4A, CD14, CD36, CRP, GAS6, GSN, HNRNPC, IGHG2, IGKC, IGLC1, ITGB1, ITGB3, PYCARD, RAB7A, RAC1, THBS1]
GO:0031589	cell-substrate adhesion	GO_BiologicalProcess-EBI-UniProt-GOA-ACAP-ARAP_25.05.2022_00h00	0.00	0.00	0.00	0.00	[3]	Group15	4.91	19.00	[ACTN1, APOA1, CD36, FGA, FGB, FGG, FLNA, GAS6, ILK, ITGA2B, ITGB1, ITGB3, P4HB, PARVG, RAC1, RSU1, THBS1, VCL, VWF]

GO:0031589	cell-substrate adhesion	GO_BiologicalProcess-EBI-UniProt-GOA-ACAP-ARAP_25.05.2022_00h00	0.00	0.00	0.00	0.00	[3]	Group20	4.91	19.00	[ACTN1, APOA1, CD36, FGA, FGB, FGG, FLNA, GAS6, ILK, ITGA2B, ITGB1, ITGB3, P4HB, PARVG, RAC1, RSU1, THBS1, VCL, VWF]
GO:0006959	humoral immune response	GO_BiologicalProcess-EBI-UniProt-GOA-ACAP-ARAP_25.05.2022_00h00	0.00	0.00	0.00	0.00	[3]	Group16	4.82	17.00	[A2M, C1QC, C3, C4A, C7, CXCL6, DEFA1, FGA, FGB, GAPDH, HNRNPC, IGHG2, IGKC, IGLC1, LYZ, RNASE2, S100A9]
GO:0006959	humoral immune response	GO_BiologicalProcess-EBI-UniProt-GOA-ACAP-ARAP_25.05.2022_00h00	0.00	0.00	0.00	0.00	[3]	Group19	4.82	17.00	[A2M, C1QC, C3, C4A, C7, CXCL6, DEFA1, FGA, FGB, GAPDH, HNRNPC, IGHG2, IGKC, IGLC1, LYZ, RNASE2, S100A9]
GO:0042470	melanosome	GO_CellularComponent-EBI-UniProt-GOA-ACAP-ARAP_25.05.2022_00h00	0.00	0.00	0.00	0.00	[5, 6, 8]	Group01	13.56	16.00	[ABCB6, ANXA2, ANXA2P2, ANXA6, CAPG, CCT4, GANAB, GSN, ITGB1, ITGB3, P4HB, RAB27B, RAB7A, RAC1, STOM, YWHAB]
GO:0007596	blood coagulation	GO_BiologicalProcess-EBI-UniProt-GOA-ACAP-ARAP_25.05.2022_00h00	0.00	0.00	0.00	0.00	[3, 5]	Group15	6.02	15.00	[ACTN1, ANXA2, CD36, FGA, FGB, FGG, FLNA, GAS6, HSPB1, ILK, ITGB3, PPIA, THBS1, VCL, VWF]
GO:0045296	cadherin binding	GO_MolecularFunction-EBI-UniProt-GOA-ACAP-ARAP_25.05.2022_00h00	0.00	0.00	0.00	0.00	[4]	Group15	4.19	15.00	[ABI1, ALDOA, ANXA1, ANXA2, CAPG, CDH1, EEF1D, FLNA, ITGB1, PFN1, PRDX6, SHTN1, TAGLN2, VCL, YWHAB]
GO:0007596	blood coagulation	GO_BiologicalProcess-EBI-UniProt-GOA-ACAP-ARAP_25.05.2022_00h00	0.00	0.00	0.00	0.00	[3, 5]	Group20	6.02	15.00	[ACTN1, ANXA2, CD36, FGA, FGB, FGG, FLNA, GAS6, HSPB1, ILK, ITGB3, PPIA, THBS1, VCL, VWF]
GO:0031091	platelet alpha granule	GO_CellularComponent-EBI-UniProt-GOA-ACAP-ARAP_25.05.2022_00h00	0.00	0.00	0.00	0.00	[4, 6, 7, 9]	Group20	15.31	15.00	[A2M, ACTN1, ALDOA, APP, CD36, FGA, FGB, FGG, GAS6, ITGA2B, ITGB3, THBS1, TIMP1, TOR4A, VWF]
GO:0007160	cell-matrix adhesion	GO_BiologicalProcess-EBI-UniProt-GOA-ACAP-ARAP_25.05.2022_00h00	0.00	0.00	0.00	0.00	[4]	Group20	5.20	13.00	[ACTN1, CD36, FGA, FGB, FGG, ILK, ITGA2B, ITGB1, ITGB3, PARVG, RAC1, THBS1, VCL]
GO:0010810	regulation of cell-substrate adhesion	GO_BiologicalProcess-EBI-UniProt-GOA-ACAP-ARAP_25.05.2022_00h00	0.00	0.00	0.00	0.00	[4, 5]	Group20	5.60	13.00	[APOA1, CD36, FGA, FGB, FGG, FLNA, ILK, ITGB3, P4HB, RAC1, RSU1, THBS1, VCL]
GO:0007160	cell-matrix adhesion	GO_BiologicalProcess-EBI-UniProt-GOA-ACAP-ARAP_25.05.2022_00h00	0.00	0.00	0.00	0.00	[4]	Group21	5.20	13.00	[ACTN1, CD36, FGA, FGB, FGG, ILK, ITGA2B, ITGB1, ITGB3, PARVG, RAC1, THBS1, VCL]
GO:0031093	platelet alpha granule lumen	GO_CellularComponent-EBI-UniProt-GOA-ACAP-ARAP_25.05.2022_00h00	0.00	0.00	0.00	0.00	[5, 6, 7, 8, 9, 1	Group09	16.44	12.00	[A2M, ACTN1, ALDOA, APP, FGA, FGB, FGG, GAS6, THBS1, TIMP1, TOR4A, VWF]

GO:0031093	platelet alpha granule lumen	GO_CellularComponent-EBI-UniProt-GOA-ACAP-ARAP_25.05.2022_00h00	0.00	0.00	0.00	0.00	[5, 6, 7, 8, 9, [illegible]	Group20	16.44	12.00	[A2M, ACTN1, ALDOA, APP, FGA, FGB, FGG, GAS6, THBS1, TIMP1, TOR4A, VWF]
GO:0005775	vacuolar lumen	GO_CellularComponent-EBI-UniProt-GOA-ACAP-ARAP_25.05.2022_00h00	0.00	0.00	0.00	0.00	[4, 5, 6]	Group04	5.58	11.00	[ANXA2, C3, CPPED1, DEFA1, GC, IFI30, LYZ, MNDA, PRDX6, PYCARD, RNASE2]
GO:0061134	peptidase regulator activity	GO_BiologicalProcess-EBI-UniProt-GOA-ACAP-ARAP_25.05.2022_00h00	0.00	0.00	0.00	0.00	[5, 6, 7, 8]	Group06	4.47	11.00	[A2M, ANXA2, APP, C3, C4A, GAPDH, GAS6, PYCARD, SPP2, TIMP1, VSIR]
GO:0010951	negative regulation of endopeptidase activity	GO_BiologicalProcess-EBI-UniProt-GOA-ACAP-ARAP_25.05.2022_00h00	0.00	0.00	0.00	0.00	[7, 8, 9]	Group06	4.10	11.00	[A2M, ANXA2, APP, C3, C4A, GAPDH, GAS6, GPX1, SPP2, THBS1, TIMP1]
GO:0030168	platelet activation	GO_BiologicalProcess-EBI-UniProt-GOA-ACAP-ARAP_25.05.2022_00h00	0.00	0.00	0.00	0.00	[3, 4, 6]	Group15	7.48	11.00	[ACTN1, FGA, FGB, FGG, FLNA, HSPB1, ILK, ITGB3, PPIA, VCL, VWF]
GO:0030168	platelet activation	GO_BiologicalProcess-EBI-UniProt-GOA-ACAP-ARAP_25.05.2022_00h00	0.00	0.00	0.00	0.00	[3, 4, 6]	Group20	7.48	11.00	[ACTN1, FGA, FGB, FGG, FLNA, HSPB1, ILK, ITGB3, PPIA, VCL, VWF]
GO:0010811	positive regulation of cell-substrate adhesion	GO_BiologicalProcess-EBI-UniProt-GOA-ACAP-ARAP_25.05.2022_00h00	0.00	0.00	0.00	0.00	[4, 5, 6]	Group20	8.09	11.00	[APOA1, CD36, FGA, FGB, FGG, FLNA, ILK, ITGB3, P4HB, RAC1, RSU1]
GO:0042582	azurophil granule	GO_CellularComponent-EBI-UniProt-GOA-ACAP-ARAP_25.05.2022_00h00	0.00	0.00	0.00	0.00	[4, 6, 7, 8, 9]	Group04	5.75	10.00	[ANXA2, C3, CPPED1, DEFA1, LYZ, MNDA, PRDX6, PYCARD, RNASE2, STOM]
GO:0101002	ficolin-1-rich granule	GO_CellularComponent-EBI-UniProt-GOA-ACAP-ARAP_25.05.2022_00h00	0.00	0.00	0.00	0.00	[4, 6, 7, 9]	Group07	4.85	10.00	[ALDOA, GSN, MNDA, PNP, PPIA, PSMC2, RAC1, S100A9, SLC2A3, VCL]
GO:0034446	substrate adhesion-dependent cell spreading	GO_BiologicalProcess-EBI-UniProt-GOA-ACAP-ARAP_25.05.2022_00h00	0.00	0.00	0.00	0.00	[4, 5, 6]	Group20	8.40	10.00	[APOA1, FGA, FGB, FGG, FLNA, ILK, ITGB3, P4HB, PARVG, RAC1]
GO:1905952	regulation of lipid localization	GO_BiologicalProcess-EBI-UniProt-GOA-ACAP-ARAP_25.05.2022_00h00	0.00	0.00	0.00	0.00	[3, 4]	Group19	5.00	9.00	[ANXA2, APOA1, APOA2, C3, CD36, CRP, ITGB3, S100A9, THBS1]
GO:0006956	complement activation	GO_BiologicalProcess-EBI-UniProt-GOA-ACAP-ARAP_25.05.2022_00h00	0.00	0.00	0.00	0.00	[3, 4, 5, 6, 7]	Group19	5.81	9.00	[A2M, C1QC, C3, C4A, C7, HNRNPC, IGHG2, IGKC, IGLC1]

GO:0043277	apoptotic cell clearance	GO_BiologicalProcess-EBI-UniProt-GOA-ACAP-ARAP_25.05.2022_00h00	0.00	0.00	0.00	0.00	[6]	Group19	16.67	9.00	[ANXA1, C3, C4A, CD36, GAS6, HNRNPC, ITGB3, RAC1, THBS1]
GO:1903034	regulation of response to wounding	GO_BiologicalProcess-EBI-UniProt-GOA-ACAP-ARAP_25.05.2022_00h00	0.00	0.00	0.00	0.00	[4, 5]	Group20	5.14	9.00	[ANXA1, ANXA2, CD36, FGA, FGB, FGG, FLNA, ITGB1, THBS1]
GO:1900026	positive regulation of substrate adhesion-dependent cell spreading	GO_BiologicalProcess-EBI-UniProt-GOA-ACAP-ARAP_25.05.2022_00h00	0.00	0.00	0.00	0.00	[5, 6, 7, 8]	Group20	18.37	9.00	[APOA1, FGA, FGB, FGG, FLNA, ILK, ITGB3, P4HB, RAC1]
GO:1905952	regulation of lipid localization	GO_BiologicalProcess-EBI-UniProt-GOA-ACAP-ARAP_25.05.2022_00h00	0.00	0.00	0.00	0.00	[3, 4]	Group21	5.00	9.00	[ANXA2, APOA1, APOA2, C3, CD36, CRP, ITGB3, S100A9, THBS1]
GO:0071674	mononuclear cell migration	GO_BiologicalProcess-EBI-UniProt-GOA-ACAP-ARAP_25.05.2022_00h00	0.00	0.00	0.00	0.00	[3, 5]	Group21	4.11	9.00	[ANXA1, APP, CCL23, DEFA1, GAS6, ITGB3, PYCARD, THBS1, WNK1]
GO:1903034	regulation of response to wounding	GO_BiologicalProcess-EBI-UniProt-GOA-ACAP-ARAP_25.05.2022_00h00	0.00	0.00	0.00	0.00	[4, 5]	Group21	5.14	9.00	[ANXA1, ANXA2, CD36, FGA, FGB, FGG, FLNA, ITGB1, THBS1]
GO:0032640	tumor necrosis factor production	GO_BiologicalProcess-EBI-UniProt-GOA-ACAP-ARAP_25.05.2022_00h00	0.00	0.00	0.00	0.00	[4, 7]	Group21	5.03	9.00	[APP, CD14, CD36, GAS6, HSPB1, PYCARD, S100A9, THBS1, VSIR]
GO:0043277	apoptotic cell clearance	GO_BiologicalProcess-EBI-UniProt-GOA-ACAP-ARAP_25.05.2022_00h00	0.00	0.00	0.00	0.00	[6]	Group21	16.67	9.00	[ANXA1, C3, C4A, CD36, GAS6, HNRNPC, ITGB3, RAC1, THBS1]
GO:0032680	regulation of tumor necrosis factor production	GO_BiologicalProcess-EBI-UniProt-GOA-ACAP-ARAP_25.05.2022_00h00	0.00	0.00	0.00	0.00	[5, 6, 7, 8]	Group21	5.03	9.00	[APP, CD14, CD36, GAS6, HSPB1, PYCARD, S100A9, THBS1, VSIR]
GO:1904813	ficolin-1-rich granule lumen	GO_CellularComponent-EBI-UniProt-GOA-ACAP-ARAP_25.05.2022_00h00	0.00	0.00	0.00	0.00	[5, 7, 8, 10]	Group07	5.71	8.00	[ALDOA, GSN, MNDA, PNP, PPIA, PSMC2, S100A9, VCL]
GO:2001242	regulation of intrinsic apoptotic signaling pathway	GO_BiologicalProcess-EBI-UniProt-GOA-ACAP-ARAP_25.05.2022_00h00	0.00	0.00	0.00	0.00	[5, 6, 7, 8]	Group13	4.42	8.00	[GPX1, HSPB1, P4HB, PPIA, PYCARD, S100A9, SOD2, TIMP1]
GO:0005178	integrin binding	GO_MolecularFunction-EBI-UniProt-GOA-ACAP-ARAP_25.05.2022_00h00	0.00	0.00	0.00	0.00	[3, 4]	Group15	4.76	8.00	[ACTN1, ITGA2B, ITGB1, ITGB3, P4HB, PPIA, THBS1, VWF]

GO:0019730	antimicrobial humoral response	GO_BiologicalProcess-EBI-UniProt-GOA-ACAP-ARAP_25.05.2022_00h00	0.00	0.00	0.00	0.00	[4, 5, 6]	Group16	6.02	8.00	[CXCL6, DEFA1, FGA, FGB, GAPDH, LYZ, RNASE2, S100A9]
GO:0019730	antimicrobial humoral response	GO_BiologicalProcess-EBI-UniProt-GOA-ACAP-ARAP_25.05.2022_00h00	0.00	0.00	0.00	0.00	[4, 5, 6]	Group19	6.02	8.00	[CXCL6, DEFA1, FGA, FGB, GAPDH, LYZ, RNASE2, S100A9]
GO:0050766	positive regulation of phagocytosis	GO_BiologicalProcess-EBI-UniProt-GOA-ACAP-ARAP_25.05.2022_00h00	0.00	0.00	0.00	0.00	[3, 4, 5, 6, 7]	Group19	10.00	8.00	[APOA1, APOA2, C3, C4A, CD36, GAS6, HNRNPC, PYCARD]
GO:0006911	phagocytosis, engulfment	GO_BiologicalProcess-EBI-UniProt-GOA-ACAP-ARAP_25.05.2022_00h00	0.00	0.00	0.00	0.00	[6, 7]	Group19	5.33	8.00	[C3, CD36, GSN, IGHG2, IGKC, IGLC1, RAC1, THBS1]
GO:0005178	integrin binding	GO_MolecularFunction-EBI-UniProt-GOA-ACAP-ARAP_25.05.2022_00h00	0.00	0.00	0.00	0.00	[3, 4]	Group20	4.76	8.00	[ACTN1, ITGA2B, ITGB1, ITGB3, P4HB, PPIA, THBS1, VWF]
GO:0019730	antimicrobial humoral response	GO_BiologicalProcess-EBI-UniProt-GOA-ACAP-ARAP_25.05.2022_00h00	0.00	0.00	0.00	0.00	[4, 5, 6]	Group20	6.02	8.00	[CXCL6, DEFA1, FGA, FGB, GAPDH, LYZ, RNASE2, S100A9]
GO:0005178	integrin binding	GO_MolecularFunction-EBI-UniProt-GOA-ACAP-ARAP_25.05.2022_00h00	0.00	0.00	0.00	0.00	[3, 4]	Group21	4.76	8.00	[ACTN1, ITGA2B, ITGB1, ITGB3, P4HB, PPIA, THBS1, VWF]
GO:0032612	interleukin-1 production	GO_BiologicalProcess-EBI-UniProt-GOA-ACAP-ARAP_25.05.2022_00h00	0.00	0.00	0.00	0.00	[3, 6]	Group21	6.50	8.00	[ANXA1, APOA1, APP, CD36, GAS6, HSPB1, MNDA, PYCARD]
GO:0050766	positive regulation of phagocytosis	GO_BiologicalProcess-EBI-UniProt-GOA-ACAP-ARAP_25.05.2022_00h00	0.00	0.00	0.00	0.00	[3, 4, 5, 6, 7]	Group21	10.00	8.00	[APOA1, APOA2, C3, C4A, CD36, GAS6, HNRNPC, PYCARD]
GO:0032652	regulation of interleukin-1 production	GO_BiologicalProcess-EBI-UniProt-GOA-ACAP-ARAP_25.05.2022_00h00	0.00	0.00	0.00	0.00	[4, 5, 6, 7]	Group21	6.50	8.00	[ANXA1, APOA1, APP, CD36, GAS6, HSPB1, MNDA, PYCARD]
GO:0071621	granulocyte chemotaxis	GO_BiologicalProcess-EBI-UniProt-GOA-ACAP-ARAP_25.05.2022_00h00	0.00	0.00	0.00	0.00	[4, 5, 6, 7]	Group11	4.90	7.00	[ANXA1, CCL23, CXCL6, PPIA, RAC1, S100A9, THBS1]
GO:0002687	positive regulation of leukocyte migration	GO_BiologicalProcess-EBI-UniProt-GOA-ACAP-ARAP_25.05.2022_00h00	0.00	0.01	0.00	0.00	[3, 4, 5, 6, 7]	Group19	4.40	7.00	[APP, GAS6, ITGB3, PYCARD, RAC1, THBS1, WNK1]

GO:0031638	zymogen activation	GO_BiologicalProcess-EBI-UniProt-GOA-ACAP-ARAP_25.05.2022_00h00	0.00	0.00	0.00	0.00	[6, 7]	Group20	9.86	7.00	[ANXA2, FGA, FGB, FGG, PYCARD, THBS1, VSIR]
GO:0019955	cytokine binding	GO_MolecularFunction-EBI-UniProt-GOA-ACAP-ARAP_25.05.2022_00h00	0.00	0.01	0.00	0.00	[3]	Group21	4.49	7.00	[A2M, CCL23, CD36, ITGB1, ITGB3, TGFBR3, THBS1]
GO:0002687	positive regulation of leukocyte migration	GO_BiologicalProcess-EBI-UniProt-GOA-ACAP-ARAP_25.05.2022_00h00	0.00	0.01	0.00	0.00	[3, 4, 5, 6, 7]	Group21	4.40	7.00	[APP, GAS6, ITGB3, PYCARD, RAC1, THBS1, WNK1]
GO:0072676	lymphocyte migration	GO_BiologicalProcess-EBI-UniProt-GOA-ACAP-ARAP_25.05.2022_00h00	0.00	0.00	0.00	0.00	[4, 6]	Group21	5.38	7.00	[APP, CCL23, DEFA1, GAS6, ITGB3, PYCARD, WNK1]
GO:0071621	granulocyte chemotaxis	GO_BiologicalProcess-EBI-UniProt-GOA-ACAP-ARAP_25.05.2022_00h00	0.00	0.00	0.00	0.00	[4, 5, 6, 7]	Group21	4.90	7.00	[ANXA1, CCL23, CXCL6, PPIA, RAC1, S100A9, THBS1]
GO:0032760	positive regulation of tumor necrosis factor production	GO_BiologicalProcess-EBI-UniProt-GOA-ACAP-ARAP_25.05.2022_00h00	0.00	0.00	0.00	0.00	[5, 6, 7, 8, 9]	Group21	6.14	7.00	[APP, CD14, CD36, HSPB1, PYCARD, S100A9, THBS1]
GO:1990748	cellular detoxification	GO_BiologicalProcess-EBI-UniProt-GOA-ACAP-ARAP_25.05.2022_00h00	0.00	0.01	0.00	0.00	[2, 5]	Group13	4.55	6.00	[ABCB6, CD36, GPX1, PRDX6, S100A9, SOD2]
GO:1900407	regulation of cellular response to oxidative stress	GO_BiologicalProcess-EBI-UniProt-GOA-ACAP-ARAP_25.05.2022_00h00	0.00	0.00	0.00	0.00	[4, 5, 6]	Group13	6.45	6.00	[CD36, GPX1, HSPB1, P4HB, PPIA, SOD2]
GO:2001243	negative regulation of intrinsic apoptotic signaling pathway	GO_BiologicalProcess-EBI-UniProt-GOA-ACAP-ARAP_25.05.2022_00h00	0.00	0.01	0.00	0.00	[5, 6, 7, 8, 9]	Group13	5.26	6.00	[GPX1, HSPB1, PPIA, S100A9, SOD2, TIMP1]
GO:0030193	regulation of blood coagulation	GO_BiologicalProcess-EBI-UniProt-GOA-ACAP-ARAP_25.05.2022_00h00	0.00	0.00	0.00	0.00	[3, 4, 5, 6, 7]	Group18	7.59	6.00	[ANXA2, CD36, FGA, FGB, FGG, THBS1]
GO:0032368	regulation of lipid transport	GO_BiologicalProcess-EBI-UniProt-GOA-ACAP-ARAP_25.05.2022_00h00	0.00	0.02	0.00	0.00	[4, 5, 6]	Group18	4.05	6.00	[ANXA2, APOA1, APOA2, ITGB3, S100A9, THBS1]
GO:1904019	epithelial cell apoptotic process	GO_BiologicalProcess-EBI-UniProt-GOA-ACAP-ARAP_25.05.2022_00h00	0.00	0.01	0.00	0.00	[5]	Group18	4.48	6.00	[FGA, FGB, FGG, GAS6, GSN, THBS1]

GO:1904035	regulation of epithelial cell apoptotic process	GO_BiologicalProcess-EBI-UniProt-GOA-ACAP-ARAP_25.05.2022_00h00	0.00	0.01	0.00	0.00	[6, 7]	Group18	5.66	6.00	[FGA, FGB, FGG, GAS6, GSN, THBS1]
GO:0030193	regulation of blood coagulation	GO_BiologicalProcess-EBI-UniProt-GOA-ACAP-ARAP_25.05.2022_00h00	0.00	0.00	0.00	0.00	[3, 4, 5, 6, 7]	Group20	7.59	6.00	[ANXA2, CD36, FGA, FGB, FGG, THBS1]
GO:1904019	epithelial cell apoptotic process	GO_BiologicalProcess-EBI-UniProt-GOA-ACAP-ARAP_25.05.2022_00h00	0.00	0.01	0.00	0.00	[5]	Group20	4.48	6.00	[FGA, FGB, FGG, GAS6, GSN, THBS1]
GO:1904035	regulation of epithelial cell apoptotic process	GO_BiologicalProcess-EBI-UniProt-GOA-ACAP-ARAP_25.05.2022_00h00	0.00	0.01	0.00	0.00	[6, 7]	Group20	5.66	6.00	[FGA, FGB, FGG, GAS6, GSN, THBS1]
GO:0019838	growth factor binding	GO_MolecularFunction-EBI-UniProt-GOA-ACAP-ARAP_25.05.2022_00h00	0.00	0.02	0.00	0.00	[3]	Group21	4.26	6.00	[A2M, CD36, IGFBP2, ITGB3, TGFBR3, THBS1]
GO:0030193	regulation of blood coagulation	GO_BiologicalProcess-EBI-UniProt-GOA-ACAP-ARAP_25.05.2022_00h00	0.00	0.00	0.00	0.00	[3, 4, 5, 6, 7]	Group21	7.59	6.00	[ANXA2, CD36, FGA, FGB, FGG, THBS1]
GO:0032368	regulation of lipid transport	GO_BiologicalProcess-EBI-UniProt-GOA-ACAP-ARAP_25.05.2022_00h00	0.00	0.02	0.00	0.00	[4, 5, 6]	Group21	4.05	6.00	[ANXA2, APOA1, APOA2, ITGB3, S100A9, THBS1]
GO:0032611	interleukin-1 beta production	GO_BiologicalProcess-EBI-UniProt-GOA-ACAP-ARAP_25.05.2022_00h00	0.00	0.01	0.00	0.00	[4, 7]	Group21	5.83	6.00	[APOA1, APP, CD36, HSPB1, MNDA, PYCARD]
GO:0071675	regulation of mononuclear cell migration	GO_BiologicalProcess-EBI-UniProt-GOA-ACAP-ARAP_25.05.2022_00h00	0.00	0.01	0.00	0.00	[4, 5, 6, 7]	Group21	4.65	6.00	[APP, GAS6, ITGB3, PYCARD, THBS1, WNK1]
GO:0032651	regulation of interleukin-1 beta production	GO_BiologicalProcess-EBI-UniProt-GOA-ACAP-ARAP_25.05.2022_00h00	0.00	0.01	0.00	0.00	[5, 6, 7, 8]	Group21	5.83	6.00	[APOA1, APP, CD36, HSPB1, MNDA, PYCARD]
GO:0043534	blood vessel endothelial cell migration	GO_BiologicalProcess-EBI-UniProt-GOA-ACAP-ARAP_25.05.2022_00h00	0.00	0.01	0.00	0.00	[6, 7]	Group21	4.80	6.00	[ANXA1, APOA1, GPX1, HSPB1, ITGB1, THBS1]
GO:0046822	regulation of nucleocytoplasmic transport	GO_BiologicalProcess-EBI-UniProt-GOA-ACAP-ARAP_25.05.2022_00h00	0.00	0.03	0.00	0.00	[5, 6, 7]	Group03	4.24	5.00	[CD36, CDH1, FLNA, GAS6, KHDRBS1]

GO:0045185	maintenance of protein location	GO_BiologicalProcess-EBI-UniProt-GOA-ACAP-ARAP_25.05.2022_00h00	0.00	0.02	0.00	0.00	[3, 5]	Group05	4.90	5.00	[FLNA, GSN, HNRNPU, KDELR1, YWHAB]
GO:0032432	actin filament bundle	GO_CellularComponent-EBI-UniProt-GOA-ACAP-ARAP_25.05.2022_00h00	0.00	0.02	0.00	0.00	[2, 7]	Group08	5.81	5.00	[ACTN1, FLNA, ILK, MYH14, TPM4]
GO:0032677	regulation of interleukin-8 production	GO_BiologicalProcess-EBI-UniProt-GOA-ACAP-ARAP_25.05.2022_00h00	0.00	0.02	0.00	0.00	[4, 5, 6, 7]	Group10	5.32	5.00	[ANXA1, APOA2, CD14, CRP, PYCARD]
GO:0010595	positive regulation of endothelial cell migration	GO_BiologicalProcess-EBI-UniProt-GOA-ACAP-ARAP_25.05.2022_00h00	0.00	0.03	0.00	0.00	[5, 6, 7, 8]	Group11	4.24	5.00	[ANXA1, HSPB1, ITGB3, RAC1, THBS1]
GO:0030593	neutrophil chemotaxis	GO_BiologicalProcess-EBI-UniProt-GOA-ACAP-ARAP_25.05.2022_00h00	0.00	0.03	0.00	0.00	[5, 6, 7, 8]	Group11	4.35	5.00	[CCL23, CXCL6, PPIA, RAC1, S100A9]
GO:0032204	regulation of telomere maintenance	GO_BiologicalProcess-EBI-UniProt-GOA-ACAP-ARAP_25.05.2022_00h00	0.00	0.03	0.00	0.00	[5, 6, 7, 8]	Group12	4.63	5.00	[CCT4, HNRNPC, HNRNPD, HNRNPU, XRCC5]
GO:0006278	RNA-templated DNA biosynthetic process	GO_BiologicalProcess-EBI-UniProt-GOA-ACAP-ARAP_25.05.2022_00h00	0.00	0.03	0.00	0.00	[6, 7, 8]	Group12	4.72	5.00	[CCT4, HNRNPC, HNRNPD, HNRNPU, XRCC5]
GO:0008631	intrinsic apoptotic signaling pathway in response to oxidative stress	GO_BiologicalProcess-EBI-UniProt-GOA-ACAP-ARAP_25.05.2022_00h00	0.00	0.00	0.00	0.00	[4, 5, 6, 7]	Group13	10.00	5.00	[GPX1, HSPB1, P4HB, PPIA, SOD2]
GO:1902175	regulation of oxidative stress-induced intrinsic apoptotic signaling pathway	GO_BiologicalProcess-EBI-UniProt-GOA-ACAP-ARAP_25.05.2022_00h00	0.00	0.00	0.00	0.00	[5, 6, 7, 8, 9]	Group13	15.15	5.00	[GPX1, HSPB1, P4HB, PPIA, SOD2]
GO:0005546	phosphatidylinositol-4,5-bisphosphate binding	GO_MolecularFunction-EBI-UniProt-GOA-ACAP-ARAP_25.05.2022_00h00	0.00	0.02	0.00	0.00	[4, 7]	Group14	5.62	5.00	[ANXA2, ANXA2P2, CAPG, GSN, PFN1]
GO:0032677	regulation of interleukin-8 production	GO_BiologicalProcess-EBI-UniProt-GOA-ACAP-ARAP_25.05.2022_00h00	0.00	0.02	0.00	0.00	[4, 5, 6, 7]	Group14	5.32	5.00	[ANXA1, APOA2, CD14, CRP, PYCARD]
GO:0051702	biological process involved in interaction with symbiont	GO_BiologicalProcess-EBI-UniProt-GOA-ACAP-ARAP_25.05.2022_00h00	0.00	0.03	0.00	0.00	[3]	Group16	4.17	5.00	[CXCL6, DEFA1, GAPDH, GPX1, STOM]

GO:0030593	neutrophil chemotaxis	GO_BiologicalProcess-EBI-UniProt-GOA-ACAP-ARAP_25.05.2022_00h00	0.00	0.03	0.00	0.00	[5, 6, 7, 8]	Group16	4.35	5.00	[CCL23, CXCL6, PPIA, RAC1, S100A9]
GO:0048145	regulation of fibroblast proliferation	GO_BiologicalProcess-EBI-UniProt-GOA-ACAP-ARAP_25.05.2022_00h00	0.00	0.02	0.00	0.00	[4, 5]	Group17	5.21	5.00	[GAS6, IFI30, ITGB3, S100A9, SOD2]
GO:0042102	positive regulation of T cell proliferation	GO_BiologicalProcess-EBI-UniProt-GOA-ACAP-ARAP_25.05.2022_00h00	0.00	0.03	0.00	0.00	[6, 7, 8, 9]	Group17	4.46	5.00	[ANXA1, IGFBP2, PNP, PYCARD, TIMP1]
GO:0005546	phosphatidylinositol-4,5-bisphosphate binding	GO_MolecularFunction-EBI-UniProt-GOA-ACAP-ARAP_25.05.2022_00h00	0.00	0.02	0.00	0.00	[4, 7]	Group18	5.62	5.00	[ANXA2, ANXA2P2, CAPG, GSN, PFN1]
GO:0042730	fibrinolysis	GO_BiologicalProcess-EBI-UniProt-GOA-ACAP-ARAP_25.05.2022_00h00	0.00	0.00	0.00	0.00	[4, 5, 6, 7, 8, 9	Group18	15.63	5.00	[ANXA2, FGA, FGB, FGG, THBS1]
GO:0031639	plasminogen activation	GO_BiologicalProcess-EBI-UniProt-GOA-ACAP-ARAP_25.05.2022_00h00	0.00	0.00	0.00	0.00	[7, 8]	Group18	17.86	5.00	[ANXA2, FGA, FGB, FGG, THBS1]
GO:0010595	positive regulation of endothelial cell migration	GO_BiologicalProcess-EBI-UniProt-GOA-ACAP-ARAP_25.05.2022_00h00	0.00	0.03	0.00	0.00	[5, 6, 7, 8]	Group19	4.24	5.00	[ANXA1, HSPB1, ITGB3, RAC1, THBS1]
GO:1903036	positive regulation of response to wounding	GO_BiologicalProcess-EBI-UniProt-GOA-ACAP-ARAP_25.05.2022_00h00	0.00	0.01	0.00	0.00	[3, 4, 5, 6]	Group20	6.49	5.00	[ANXA1, CD36, FLNA, ITGB1, THBS1]
GO:0034114	regulation of heterotypic cell-cell adhesion	GO_BiologicalProcess-EBI-UniProt-GOA-ACAP-ARAP_25.05.2022_00h00	0.00	0.00	0.00	0.00	[5, 6]	Group20	18.52	5.00	[APOA1, FGA, FGB, FGG, WNK1]
GO:0007044	cell-substrate junction assembly	GO_BiologicalProcess-EBI-UniProt-GOA-ACAP-ARAP_25.05.2022_00h00	0.00	0.03	0.00	0.00	[6]	Group20	4.72	5.00	[ACTN1, ITGB3, RAC1, THBS1, VCL]
GO:0042730	fibrinolysis	GO_BiologicalProcess-EBI-UniProt-GOA-ACAP-ARAP_25.05.2022_00h00	0.00	0.00	0.00	0.00	[4, 5, 6, 7, 8, 9	Group20	15.63	5.00	[ANXA2, FGA, FGB, FGG, THBS1]
GO:2001237	negative regulation of extrinsic apoptotic signaling pathway	GO_BiologicalProcess-EBI-UniProt-GOA-ACAP-ARAP_25.05.2022_00h00	0.00	0.03	0.00	0.00	[5, 6, 7, 8, 9]	Group20	4.63	5.00	[FGA, FGB, FGG, GPX1, THBS1]

GO:0031639	plasminogen activation	GO_BiologicalProcess-EBI-UniProt-GOA-ACAP-ARAP_25.05.2022_00h00	0.00	0.00	0.00	0.00	[7, 8]	Group20	17.86	5.00	[ANXA2, FGA, FGB, FGG, THBS1]
GO:1902041	regulation of extrinsic apoptotic signaling pathway via death domain receptors	GO_BiologicalProcess-EBI-UniProt-GOA-ACAP-ARAP_25.05.2022_00h00	0.00	0.00	0.00	0.00	[6, 7, 8, 9]	Group20	8.77	5.00	[FGA, FGB, FGG, GPX1, THBS1]
GO:0022600	digestive system process	GO_BiologicalProcess-EBI-UniProt-GOA-ACAP-ARAP_25.05.2022_00h00	0.00	0.03	0.00	0.00	[3]	Group21	4.24	5.00	[APOA1, APOA2, CD36, SCT, WNK1]
GO:1905954	positive regulation of lipid localization	GO_BiologicalProcess-EBI-UniProt-GOA-ACAP-ARAP_25.05.2022_00h00	0.00	0.03	0.00	0.00	[2, 3, 4, 5]	Group21	4.17	5.00	[ANXA2, APOA1, C3, CD36, S100A9]
GO:0071813	lipoprotein particle binding	GO_MolecularFunction-EBI-UniProt-GOA-ACAP-ARAP_25.05.2022_00h00	0.00	0.00	0.00	0.00	[4]	Group21	15.63	5.00	[APOA1, APOA2, CD36, CRP, THBS1]
GO:1903036	positive regulation of response to wounding	GO_BiologicalProcess-EBI-UniProt-GOA-ACAP-ARAP_25.05.2022_00h00	0.00	0.01	0.00	0.00	[3, 4, 5, 6]	Group21	6.49	5.00	[ANXA1, CD36, FLNA, ITGB1, THBS1]
GO:0032677	regulation of interleukin-8 production	GO_BiologicalProcess-EBI-UniProt-GOA-ACAP-ARAP_25.05.2022_00h00	0.00	0.02	0.00	0.00	[4, 5, 6, 7]	Group21	5.32	5.00	[ANXA1, APOA2, CD14, CRP, PYCARD]
GO:0007044	cell-substrate junction assembly	GO_BiologicalProcess-EBI-UniProt-GOA-ACAP-ARAP_25.05.2022_00h00	0.00	0.03	0.00	0.00	[6]	Group21	4.72	5.00	[ACTN1, ITGB3, RAC1, THBS1, VCL]
GO:0038024	cargo receptor activity	GO_BiologicalProcess-EBI-UniProt-GOA-ACAP-ARAP_25.05.2022_00h00	0.00	0.02	0.00	0.00	[6]	Group21	5.21	5.00	[ANXA2, APP, CD36, ITGB3, LGALS3BP]
GO:0048259	regulation of receptor-mediated endocytosis	GO_BiologicalProcess-EBI-UniProt-GOA-ACAP-ARAP_25.05.2022_00h00	0.00	0.03	0.00	0.00	[5, 6, 7]	Group21	4.07	5.00	[ANXA2, APP, C3, ITGB3, RAC1]
GO:0072678	T cell migration	GO_BiologicalProcess-EBI-UniProt-GOA-ACAP-ARAP_25.05.2022_00h00	0.00	0.01	0.00	0.00	[5, 7]	Group21	6.58	5.00	[APP, DEFA1, ITGB3, PYCARD, WNK1]
GO:0010595	positive regulation of endothelial cell migration	GO_BiologicalProcess-EBI-UniProt-GOA-ACAP-ARAP_25.05.2022_00h00	0.00	0.03	0.00	0.00	[5, 6, 7, 8]	Group21	4.24	5.00	[ANXA1, HSPB1, ITGB3, RAC1, THBS1]

GO:0002504	antigen processing and presentation of peptide or polysaccharide antigen via MHC class II	GO_BiologicalProcess-EBI-UniProt-GOA-ACAP-ARAP_25.05.2022_00h00	0.00	0.01	0.00	0.00	[3]	Group00	10.81	4.00	[HLA-DRA, IFI30, PYCARD, THBS1]
GO:0042827	platelet dense granule	GO_CellularComponent-EBI-UniProt-GOA-ACAP-ARAP_25.05.2022_00h00	0.00	0.00	0.00	0.00	[4, 6, 7, 9]	Group02	18.18	4.00	[FAM3C, LGALS3BP, RAB27B, SPP2]
GO:0001725	stress fiber	GO_CellularComponent-EBI-UniProt-GOA-ACAP-ARAP_25.05.2022_00h00	0.00	0.04	0.00	0.00	[3, 4, 8, 9]	Group08	5.13	4.00	[ACTN1, ILK, MYH14, TPM4]
GO:1905953	negative regulation of lipid localization	GO_BiologicalProcess-EBI-UniProt-GOA-ACAP-ARAP_25.05.2022_00h00	0.00	0.02	0.00	0.00	[2, 3, 4, 5]	Group10	8.33	4.00	[APOA2, CRP, ITGB3, THBS1]
GO:0055102	lipase inhibitor activity	GO_BiologicalProcess-EBI-UniProt-GOA-ACAP-ARAP_25.05.2022_00h00	0.00	0.00	0.00	0.00	[6]	Group10	20.00	4.00	[ANXA1, ANXA2, ANXA2P2, APOA2]
GO:0061844	antimicrobial humoral immune response mediated by antimicrobial peptide	GO_BiologicalProcess-EBI-UniProt-GOA-ACAP-ARAP_25.05.2022_00h00	0.00	0.05	0.00	0.00	[5, 6, 7]	Group11	4.65	4.00	[CXCL6, DEFA1, GAPDH, S100A9]
GO:0032210	regulation of telomere maintenance via telomerase	GO_BiologicalProcess-EBI-UniProt-GOA-ACAP-ARAP_25.05.2022_00h00	0.00	0.03	0.00	0.00	[6, 7, 8, 9, 10]	Group12	6.67	4.00	[CCT4, HNRNPC, HNRNPU, XRCC5]
GO:0034381	plasma lipoprotein particle clearance	GO_BiologicalProcess-EBI-UniProt-GOA-ACAP-ARAP_25.05.2022_00h00	0.00	0.01	0.00	0.00	[2, 3, 4]	Group14	9.30	4.00	[ANXA2, APOA1, APOA2, CD36]
GO:0055102	lipase inhibitor activity	GO_BiologicalProcess-EBI-UniProt-GOA-ACAP-ARAP_25.05.2022_00h00	0.00	0.00	0.00	0.00	[6]	Group14	20.00	4.00	[ANXA1, ANXA2, ANXA2P2, APOA2]
GO:0019731	antibacterial humoral response	GO_BiologicalProcess-EBI-UniProt-GOA-ACAP-ARAP_25.05.2022_00h00	0.00	0.03	0.00	0.00	[5, 6, 7]	Group16	5.88	4.00	[DEFA1, FGA, FGB, RNASE2]
GO:0061844	antimicrobial humoral immune response mediated by antimicrobial peptide	GO_BiologicalProcess-EBI-UniProt-GOA-ACAP-ARAP_25.05.2022_00h00	0.00	0.05	0.00	0.00	[5, 6, 7]	Group16	4.65	4.00	[CXCL6, DEFA1, GAPDH, S100A9]
GO:0016860	intramolecular oxidoreductase activity	GO_MolecularFunction-EBI-UniProt-GOA-ACAP-ARAP_25.05.2022_00h00	0.00	0.03	0.00	0.00	[3]	Group17	6.35	4.00	[ITGB3, P4HB, PDIA5, S100A9]

GO:0071470	cellular response to osmotic stress	GO_BiologicalProcess-EBI-UniProt-GOA-ACAP-ARAP_25.05.2022_00h00	0.00	0.02	0.00	0.00	[4, 5]	Group17	7.69	4.00	[PYCARD, SCN2A, TIMP1, XRCC5]
GO:0033032	regulation of myeloid cell apoptotic process	GO_BiologicalProcess-EBI-UniProt-GOA-ACAP-ARAP_25.05.2022_00h00	0.00	0.01	0.00	0.00	[6, 7]	Group17	10.26	4.00	[ANXA1, GAS6, S100A9, TIMP1]
GO:0010762	regulation of fibroblast migration	GO_BiologicalProcess-EBI-UniProt-GOA-ACAP-ARAP_25.05.2022_00h00	0.00	0.01	0.00	0.00	[5, 6]	Group19	8.89	4.00	[ITGB1, ITGB3, RAC1, THBS1]
GO:0072378	blood coagulation, fibrin clot formation	GO_BiologicalProcess-EBI-UniProt-GOA-ACAP-ARAP_25.05.2022_00h00	0.00	0.00	0.00	0.00	[3, 4, 5, 6]	Group20	22.22	4.00	[FGA, FGB, FGG, FLNA]
GO:0010762	regulation of fibroblast migration	GO_BiologicalProcess-EBI-UniProt-GOA-ACAP-ARAP_25.05.2022_00h00	0.00	0.01	0.00	0.00	[5, 6]	Group20	8.89	4.00	[ITGB1, ITGB3, RAC1, THBS1]
GO:0019731	antibacterial humoral response	GO_BiologicalProcess-EBI-UniProt-GOA-ACAP-ARAP_25.05.2022_00h00	0.00	0.03	0.00	0.00	[5, 6, 7]	Group20	5.88	4.00	[DEFA1, FGA, FGB, RNASE2]
GO:0034381	plasma lipoprotein particle clearance	GO_BiologicalProcess-EBI-UniProt-GOA-ACAP-ARAP_25.05.2022_00h00	0.00	0.01	0.00	0.00	[2, 3, 4]	Group21	9.30	4.00	[ANXA2, APOA1, APOA2, CD36]
GO:1905953	negative regulation of lipid localization	GO_BiologicalProcess-EBI-UniProt-GOA-ACAP-ARAP_25.05.2022_00h00	0.00	0.02	0.00	0.00	[2, 3, 4, 5]	Group21	8.33	4.00	[APOA2, CRP, ITGB3, THBS1]
GO:0019956	chemokine binding	GO_MolecularFunction-EBI-UniProt-GOA-ACAP-ARAP_25.05.2022_00h00	0.00	0.01	0.00	0.00	[4]	Group21	10.81	4.00	[A2M, CCL23, ITGB1, ITGB3]
GO:0010762	regulation of fibroblast migration	GO_BiologicalProcess-EBI-UniProt-GOA-ACAP-ARAP_25.05.2022_00h00	0.00	0.01	0.00	0.00	[5, 6]	Group21	8.89	4.00	[ITGB1, ITGB3, RAC1, THBS1]
GO:0048247	lymphocyte chemotaxis	GO_BiologicalProcess-EBI-UniProt-GOA-ACAP-ARAP_25.05.2022_00h00	0.00	0.03	0.00	0.00	[4, 5, 6, 7]	Group21	5.88	4.00	[CCL23, DEFA1, GAS6, WNK1]
GO:0014704	intercalated disc	GO_CellularComponent-EBI-UniProt-GOA-ACAP-ARAP_25.05.2022_00h00	0.00	0.02	0.00	0.00	[6]	Group21	7.02	4.00	[ACTN1, ITGB1, SCN2A, VCL]

GO:0055102	lipase inhibitor activity	GO_BiologicalProcess-EBI-UniProt-GOA-ACAP-ARAP_25.05.2022_00h00	0.00	0.00	0.00	0.00	[6]	Group21	20.00	4.00	[ANXA1, ANXA2, ANXA2P2, APOA2]
GO:0005041	low-density lipoprotein particle receptor activity	GO_BiologicalProcess-EBI-UniProt-GOA-ACAP-ARAP_25.05.2022_00h00	0.00	0.00	0.00	0.00	[8]	Group21	13.79	4.00	[ANXA2, APP, CD36, ITGB3]
GO:2000406	positive regulation of T cell migration	GO_BiologicalProcess-EBI-UniProt-GOA-ACAP-ARAP_25.05.2022_00h00	0.00	0.01	0.00	0.00	[6, 7, 8, 9, 10]	Group21	11.11	4.00	[APP, ITGB3, PYCARD, WNK1]
GO:0032507	maintenance of protein location in cell	GO_BiologicalProcess-EBI-UniProt-GOA-ACAP-ARAP_25.05.2022_00h00	0.01	0.01	0.00	0.00	[3, 4, 6]	Group05	4.17	3.00	[GSN, HNRNPU, KDELR1]
GO:0032757	positive regulation of interleukin-8 production	GO_BiologicalProcess-EBI-UniProt-GOA-ACAP-ARAP_25.05.2022_00h00	0.01	0.01	0.00	0.00	[4, 5, 6, 7, 8]	Group10	4.17	3.00	[APOA2, CD14, PYCARD]
GO:0032206	positive regulation of telomere maintenance	GO_BiologicalProcess-EBI-UniProt-GOA-ACAP-ARAP_25.05.2022_00h00	0.01	0.03	0.00	0.00	[5, 6, 7, 8, 9]	Group12	4.23	3.00	[CCT4, HNRNPD, XRCC5]
GO:0070613	regulation of protein processing	GO_BiologicalProcess-EBI-UniProt-GOA-ACAP-ARAP_25.05.2022_00h00	0.01	0.04	0.00	0.00	[6, 7]	Group14	4.29	3.00	[ANXA2, GSN, THBS1]
GO:0032374	regulation of cholesterol transport	GO_BiologicalProcess-EBI-UniProt-GOA-ACAP-ARAP_25.05.2022_00h00	0.01	0.05	0.00	0.00	[6, 7, 8]	Group14	4.35	3.00	[ANXA2, APOA1, APOA2]
GO:0019834	phospholipase A2 inhibitor activity	GO_BiologicalProcess-EBI-UniProt-GOA-ACAP-ARAP_25.05.2022_00h00	0.00	0.00	0.00	0.00	[8]	Group14	50.00	3.00	[ANXA1, ANXA2, ANXA2P2]
GO:0051873	killing by host of symbiont cells	GO_BiologicalProcess-EBI-UniProt-GOA-ACAP-ARAP_25.05.2022_00h00	0.00	0.03	0.00	0.00	[4, 5, 6]	Group16	8.82	3.00	[CXCL6, DEFA1, GAPDH]
GO:0003756	protein disulfide isomerase activity	GO_MolecularFunction-EBI-UniProt-GOA-ACAP-ARAP_25.05.2022_00h00	0.00	0.02	0.00	0.00	[3, 5]	Group17	15.00	3.00	[ITGB3, P4HB, PDIA5]
GO:0042104	positive regulation of activated T cell proliferation	GO_BiologicalProcess-EBI-UniProt-GOA-ACAP-ARAP_25.05.2022_00h00	0.00	0.03	0.00	0.00	[7, 8, 9, 10]	Group17	10.34	3.00	[IGFBP2, PYCARD, TIMP1]

GO:0070613	regulation of protein processing	GO_BiologicalProcess-EBI-UniProt-GOA-ACAP-ARAP_25.05.2022_00h00	0.01	0.04	0.00	0.00	[6, 7]	Group18	4.29	3.00	[ANXA2, GSN, THBS1]
GO:2000427	positive regulation of apoptotic cell clearance	GO_BiologicalProcess-EBI-UniProt-GOA-ACAP-ARAP_25.05.2022_00h00	0.00	0.00	0.00	0.00	[4, 5, 6, 7, 8]	Group19	33.33	3.00	[C3, C4A, HNRNPC]
GO:0050431	transforming growth factor beta binding	GO_MolecularFunction-EBI-UniProt-GOA-ACAP-ARAP_25.05.2022_00h00	0.00	0.03	0.00	0.00	[4]	Group20	11.11	3.00	[CD36, TGFBR3, THBS1]
GO:0030169	low-density lipoprotein particle binding	GO_MolecularFunction-EBI-UniProt-GOA-ACAP-ARAP_25.05.2022_00h00	0.00	0.01	0.00	0.00	[5]	Group20	15.79	3.00	[CD36, CRP, THBS1]
GO:0034116	positive regulation of heterotypic cell-cell adhesion	GO_BiologicalProcess-EBI-UniProt-GOA-ACAP-ARAP_25.05.2022_00h00	0.00	0.01	0.00	0.00	[5, 6, 7]	Group20	20.00	3.00	[FGA, FGB, FGG]
GO:0045907	positive regulation of vasoconstriction	GO_BiologicalProcess-EBI-UniProt-GOA-ACAP-ARAP_25.05.2022_00h00	0.00	0.04	0.00	0.00	[3, 4, 5, 6, 7, 8	Group20	7.89	3.00	[FGA, FGB, FGG]
GO:0070613	regulation of protein processing	GO_BiologicalProcess-EBI-UniProt-GOA-ACAP-ARAP_25.05.2022_00h00	0.01	0.04	0.00	0.00	[6, 7]	Group20	4.29	3.00	[ANXA2, GSN, THBS1]
GO:0017134	fibroblast growth factor binding	GO_MolecularFunction-EBI-UniProt-GOA-ACAP-ARAP_25.05.2022_00h00	0.00	0.02	0.00	0.00	[4]	Group21	13.04	3.00	[ITGB3, TGFBR3, THBS1]
GO:0050431	transforming growth factor beta binding	GO_MolecularFunction-EBI-UniProt-GOA-ACAP-ARAP_25.05.2022_00h00	0.00	0.03	0.00	0.00	[4]	Group21	11.11	3.00	[CD36, TGFBR3, THBS1]
GO:0008035	high-density lipoprotein particle binding	GO_MolecularFunction-EBI-UniProt-GOA-ACAP-ARAP_25.05.2022_00h00	0.00	0.00	0.00	0.00	[5]	Group21	27.27	3.00	[APOA1, APOA2, CD36]
GO:0010742	macrophage derived foam cell differentiation	GO_BiologicalProcess-EBI-UniProt-GOA-ACAP-ARAP_25.05.2022_00h00	0.00	0.05	0.00	0.00	[5]	Group21	7.32	3.00	[CD36, CRP, ITGB3]
GO:0030169	low-density lipoprotein particle binding	GO_MolecularFunction-EBI-UniProt-GOA-ACAP-ARAP_25.05.2022_00h00	0.00	0.01	0.00	0.00	[5]	Group21	15.79	3.00	[CD36, CRP, THBS1]

GO:0050996	positive regulation of lipid catabolic process	GO_BiologicalProcess-EBI-UniProt-GOA-ACAP-ARAP_25.05.2022_00h00	0.00	0.03	0.00	0.00	[4, 5, 6, 7]	Group21	10.00	3.00	[APOA1, APOA2, SCT]
GO:2000427	positive regulation of apoptotic cell clearance	GO_BiologicalProcess-EBI-UniProt-GOA-ACAP-ARAP_25.05.2022_00h00	0.00	0.00	0.00	0.00	[4, 5, 6, 7, 8]	Group21	33.33	3.00	[C3, C4A, HNRNPC]
GO:0030299	intestinal cholesterol absorption	GO_BiologicalProcess-EBI-UniProt-GOA-ACAP-ARAP_25.05.2022_00h00	0.00	0.02	0.00	0.00	[4, 6, 7, 8]	Group21	14.29	3.00	[APOA1, APOA2, CD36]
GO:0070613	regulation of protein processing	GO_BiologicalProcess-EBI-UniProt-GOA-ACAP-ARAP_25.05.2022_00h00	0.01	0.04	0.00	0.00	[6, 7]	Group21	4.29	3.00	[ANXA2, GSN, THBS1]
GO:0032374	regulation of cholesterol transport	GO_BiologicalProcess-EBI-UniProt-GOA-ACAP-ARAP_25.05.2022_00h00	0.01	0.05	0.00	0.00	[6, 7, 8]	Group21	4.35	3.00	[ANXA2, APOA1, APOA2]
GO:0051893	regulation of focal adhesion assembly	GO_BiologicalProcess-EBI-UniProt-GOA-ACAP-ARAP_25.05.2022_00h00	0.01	0.01	0.00	0.00	[6, 7, 8]	Group21	4.17	3.00	[RAC1, THBS1, VCL]
GO:0031092	platelet alpha granule membrane	GO_CellularComponent-EBI-UniProt-GOA-ACAP-ARAP_25.05.2022_00h00	0.00	0.01	0.00	0.00	[5, 6, 7, 8, 9, 1	Group21	16.67	3.00	[CD36, ITGA2B, ITGB3]
